# Organism-specific training improves performance of linear B-cell epitope prediction

**DOI:** 10.1093/bioinformatics/btab536

**Published:** 2021-07-21

**Authors:** Jodie Ashford, João Reis-Cunha, Igor Lobo, Francisco Lobo, Felipe Campelo

**Affiliations:** Department of Computer Science, College of Engineering and Physical Sciences, Aston University, Birmingham B4 7ET, UK; Department of Preventive Veterinary Medicine, Universidade Federal de Minas Gerais, Belo Horizonte 31270-901, Brazil; Graduate Program in Genetics, Universidade Federal de Minas Gerais, Belo Horizonte 31270-901, Brazil; Department of General Biology, Universidade Federal de Minas Gerais, Belo Horizonte 31270-901, Brazil; Department of Computer Science, College of Engineering and Physical Sciences, Aston University, Birmingham B4 7ET, UK

## Abstract

**Motivation:**

*In silico* identification of linear B-cell epitopes represents an important step in the development of diagnostic tests and vaccine candidates, by providing potential high-probability targets for experimental investigation. Current predictive tools were developed under a generalist approach, training models with heterogeneous datasets to develop predictors that can be deployed for a wide variety of pathogens. However, continuous advances in processing power and the increasing amount of epitope data for a broad range of pathogens indicate that training organism or taxon-specific models may become a feasible alternative, with unexplored potential gains in predictive performance.

**Results:**

This article shows how organism-specific training of epitope prediction models can yield substantial performance gains across several quality metrics when compared to models trained with heterogeneous and hybrid data, and with a variety of widely used predictors from the literature. These results suggest a promising alternative for the development of custom-tailored predictive models with high predictive power, which can be easily implemented and deployed for the investigation of specific pathogens.

**Availability and implementation:**

The data underlying this article, as well as the full reproducibility scripts, are available at https://github.com/fcampelo/OrgSpec-paper. The R package that implements the organism-specific pipeline functions is available at https://github.com/fcampelo/epitopes.

**Supplementary information:**

Supplementary materials are available at *Bioinformatics* online.

## 1 Introduction

In humoral immunity, activated B-lymphocytes (B cells) produce antibodies that bind with specific antigens, and are a key component in vertebrate immune responses (Getzoff *et al.*, 1988; Lodish *et al.*, 2000). The exact portion of an antigen that an antibody binds to is known as an *epitope* or *antigenic determinant* (Paul, 2012). Identifying B-cell epitopes is a crucial process for a number of medical and immunological processes including: vaccine development, therapeutic antibody production, disease prevention and diagnosis (Dudek *et al.*, 2010; Leinikki *et al.*, 1993; Potocnakova *et al.*, 2016).

B-cell epitopes are broadly classified into two groups: linear (or continuous) epitopes, which represent contiguous stretches of amino acid (AA) residues in an antigenic sequence; and conformational (or discontinuous) epitopes, where the AA residues that constitute these antigens are separated in the sequence and brought together by folding **(**Kindt *et al.*, 2007, Chap. 3). The methods used to predict B-cell epitopes differ depending on the type of epitope being predicted. Although the majority of B-cell epitopes are conformational (Van Regenmortel, 1996; Lo *et al.*, 2013), most epitope prediction methods are designed to predict linear epitopes (Alix, 1999; Blythe and Flower, 2005; EL-Manzalawy *et al.*, 2008; Kolaskar and Tongaonkar, 1990; Larsen *et al.*, 2006; Saha and Raghava, 2004, 2006; Singh *et al.*, 2013; Yao *et al.*, 2013). This is mainly due to a relative scarcity of available data on antigen 3D structures, as well as the high computational cost associated with predicting these structures (Yang and Yu, 2009). On the other hand, linear B-cell epitopes can be predicted from protein primary structure alone (AA sequence data), which is more readily available. Linear epitopes are also stable in a wide range of conditions, an interesting property for the transportation and storage of potential peptide vaccines. On the other hand, discontinuous epitopes can be disrupted by alterations in protein secondary/tertiary structure caused by a wide range of factors, such as variations in pH, salinity and temperature, by protein-protein interactions and post-translational modifications, among many others. In fact, linear epitopes were consistently more recognized than conformational epitopes in the sera of rabbits immunized with recombinant proteins and peptides (Forsström *et al.*, 2015). The impact of mutations is also likely to be more easily estimated for linear epitopes, where most of the relevant changes are observed in the antigenic region. Conformational epitopes, on the other hand, can be affected by AA changes in other regions of the protein that result in conformational changes, which are harder to predict and model (Pandurangan and Blundell, 2020).

Several experimental methods have been traditionally used for B-cell epitope identification, including X-ray crystallography, peptide microarrays, Western Blotting and enzyme-linked immunosorbent assay (ELISA) (Arnold *et al.*, 2018; Jespersen *et al.*, 2019). These methods are both time consuming and resource intensive, which led to the development of computational methods for epitope prediction that are commonly used as pre-screening tools for prioritizing targets for experimental investigation. Early computational methods for predicting linear epitopes were based on direct prediction of different physiochemical properties of individual AA residues found to be more represented in known epitopes, such as hydrophobicity, flexibility, surface accessibility, charge and AA residue frequency (EL-Manzalawy *et al.*, 2008; Haste Andersen *et al.*, 2006; Hopp and Woods, 1981; Parker *et al.*, 1986; Pellequer *et al.*, 1991, 1993; Yang and Yu, 2009). Numerical propensity scales are often created to represent physiochemical properties like these, and commonly used as prediction methods (Alix, 1999; Pellequer *et al.*, 1991; Pellequer and Westhof, 1993) or as input features for machine learning (ML) predictors.

Though propensity scales are still used for B-cell epitope prediction, multiple works have shown that their use alone can result in poor prediction performance (Blythe and Flower, 2005; Giacò *et al.*, 2012; Kulkarni-Kale *et al.*, 2005; Pellequer *et al.*, 1991; Ponomarenko and Bourne, 2007). This perceived limitation in predictive power, coupled with increases both in computational resources and available protein sequence data, have led to the adoption of ML models as the main methods for epitope prediction in recent years (Supplementary File S1,  Table S1-1). Several ML approaches currently exist for epitope prediction: some are trained using 3D structures, some using a combination of features from propensity scales and many more. ML methods for epitope prediction tend to outperform methods based solely on simple AA propensity scale calculations (Sanchez-Trincado *et al.*, 2017), although this is not always the case (Greenbaum *et al.*, 2007; Sanchez-Trincado *et al.*, 2017).

To our knowledge all existing epitope prediction tools are based on datasets containing labelled peptide sequences coming from a wide variety of organisms (Supplementary File S1,  Table S1-1). The use of heterogeneous datasets is associated with a common goal of developing general-purpose predictors that can be pre-trained and used out-of-the-box, without requiring users to inform the source organism of the peptides submitted for classification. In fact, as recently as 2020, Collatz *et al.* (2020) suggest that having ‘*a large variety of known epitopes from evolutionarily distinct organisms in the training set*’ would be essential to achieve bias-free classification. This is a reasonable assumption if one is aiming at developing generalist, one-size-fits-all models; however, it may be unnecessary or even counterproductive, if the new observations for which the model is expected to generalize correspond only to a specific subset of all possible observations.

Continuous advances in processing power and the increasing amount of data for distinct pathogens suggest that organism- or taxon-specific models may become a feasible alternative. Generating predictors specifically trained for individual pathogens, rather than having a single generalist model, would result in smaller but potentially higher quality training sets, resulting in better predictive performance of new epitopes for the target organism, and potentially for its phylogenetically close relatives. Under this alternative approach of training bespoke models for distinct (groups of) pathogens, the objective is to obtain predictors that generalize well only to the target organism(s), rather than to the whole variety of pathogens that may interact with a given host.

This work investigates the effects of using such organism-specific datasets to train ML models for linear B-cell epitope prediction. Proof-of-concept predictors are trained using organism-specific, heterogeneous and hybrid data, using data-rich pathogens representing two of the major classes of parasitic organisms: nematodes and viruses. The effects of these training sets on the generalization performance of the models is quantified to test whether organism-specific training can result in better predictors. The results obtained for three test cases not only support this idea, but also show that even relatively simple models trained on organism-specific data can generally outperform current state-of-the-art predictors in terms of several performance measures.

## 2 Materials and methods

**Fig. 1.**
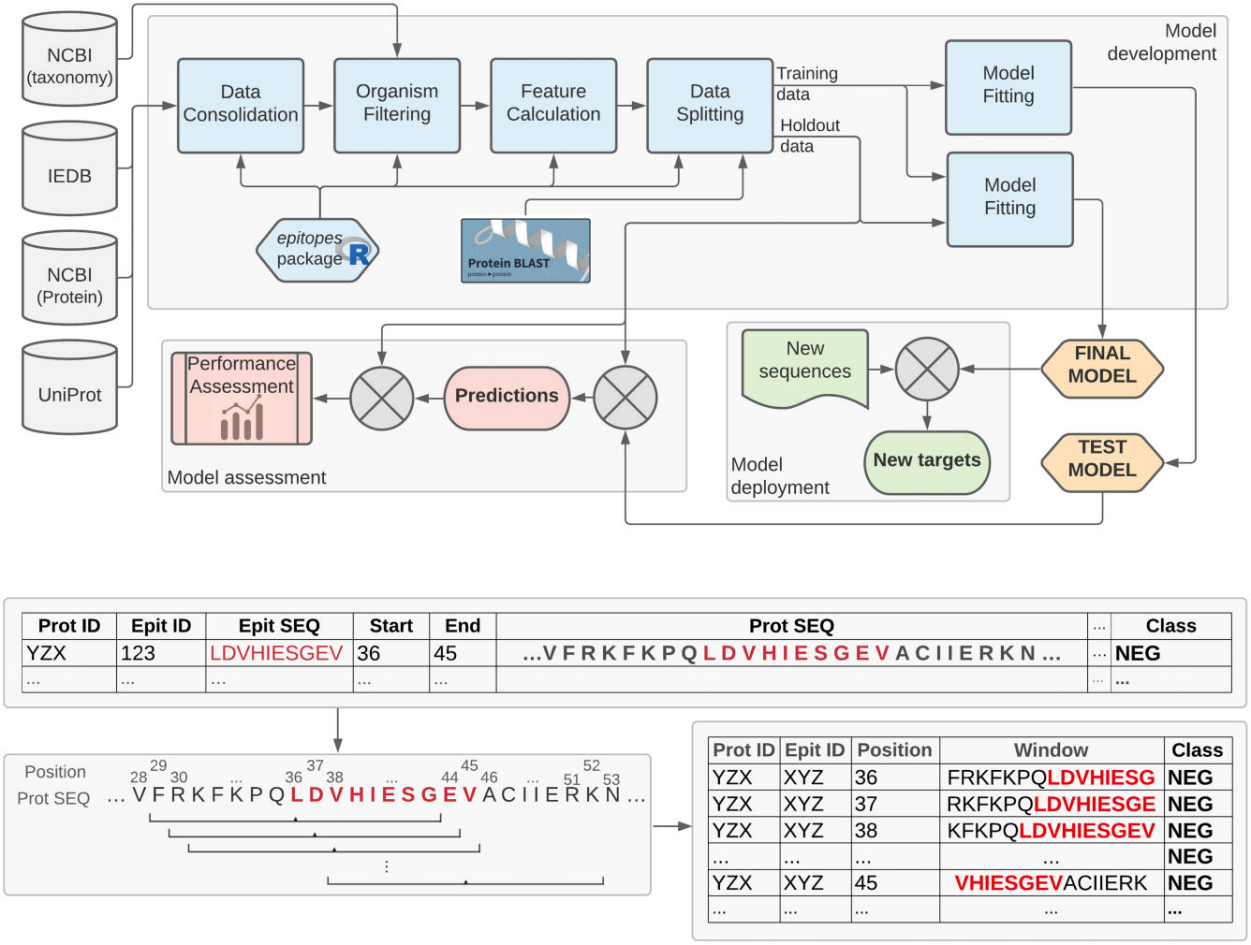
*Top*: Organism-specific epitope prediction pipeline. Publicly available data is retrieved from IEDB (Vita *et al.*, 2019), NCBI (NCBI Resource Coordinators, 2015) and UniProt (UniProt Consortium, 2020) to compose an organism-specific dataset. 845 simple features are calculated for each AA, based on the local neighbourhood of every position extracted using a 15-AA sliding window representation with a step size of one (*bottom*). The data is then split at the protein level, based on protein ID and similarity, into a training set (used for model development) and a hold-out set (used to estimate the generalization performance of the models). The *epitopes* R package, which implements the main elements of this pipeline, is available at https://fcampelo.github.io/epitopes

Figure 1 summarizes the general organism-specific epitope prediction pipeline. This section details the main methodological aspects of the proposed tool.

### 2.1 Datasets

Specific datasets for each pathogen were generated based on the full XML export of the Immune Epitope Data Base (IEDB) (Vita *et al.*, 2019) retrieved on 10 October 2020, and filtered according to the following criteria:

- Only peptides marked as linear B-cell epitopes/non-epitopes of length between 8 and 25 were selected. The filtering criteria used to isolate peptides identified as linear B-cell entries were (i) those with one or more Assays containing a ‘BCell’ field name (in the *Assay* fields of the XML document); and (ii) those containing the field ‘FragmentOfANaturalSequenceMolecule—LinearSequence’ in the *EpitopeStructure* field of the XML document. Peptides marked as either ‘Exact Epitope’ and ‘Epitope-containing region’ in the *EpitopeStructureDefines* field were included. The upper length limit was imposed to prevent overly long sequences labelled as ‘Epitope-containing region’ from adding too much noise to the training data, whereas the lower limit was set to prevent excessive redundancy due to short windows (see below, Section 2.1.1).
- Labels ‘Positive’, ‘Positive-High’, ‘Positive-Intermediate’ and ‘Positive-Low’ were grouped under the single label ‘Positive’.
- Observations with missing or inconsistent information related to the protein information (protein ID or peptide position on the protein) were removed.
- Protein information was retrieved from NCBI (NCBI Resource Coordinators, 2015) and UniProt (UniProt Consortium, 2020) based on the protein IDs available in the epitope data. Observations with invalid protein IDs were removed.
- When different assays provided conflicting evidence for the class (Positive vs. Negative) of a given peptide the class was determined by simple majority. Ties were removed from the training sets and de-classed (reference class information set as unknown) in the hold-out sets, so as not to influence the performance calculation.

For each pathogen a number of distinct datasets were generated as follows:

1. First, all examples related to the specific pathogen were extracted based on the taxonomy ID information from the IEDB data. This includes all taxonomically dependent IDs (related, e.g. to subspecies or strains) as part of the data. Prior to any data exploration or modelling, a subset of the organism-specific data consisting of approximately 25% of the available observations were set aside as a validation (*Hold-out*) set, which was not seen at any point during model construction. To minimize the chances of data leakage (Kaufman *et al.*, 2011) the splitting of the datasets was done at the protein level, based on protein ID as well as sequence coverage and similarity. Proteins with similarity and/or coverage greater than 80% were always placed within in the same split.
2. The other sub-set, containing 75% of the labelled peptides belonging to the specific pathogen, was used as the organism-specific (*OrgSpec*) training set.
3. A second training dataset (*Heterogeneous*) was assembled by random sampling of observations (grouped by taxonomy ID) from the full IEDB, *excluding any observations related to the specific pathogen*. The sampling routine included as many organisms as required to assemble a class-balanced *heterogeneous* training set containing between 2000 and 3000 labelled peptides of each class (epitope/non-epitope).
4. Finally, a third training set (*Hybrid*) was assembled by combining the *OrgSpec* and *Heterogeneous* sets.

In all cases the *Hybrid* dataset was the largest one, followed by *Heterogeneous* and then *OrgSpec*. This was set up in order to allow us to investigate the hypothesis that trading sample size (which is larger if one incorporates heterogeneous observations) by sample relevance (represented by data that belongs to the organism of interest, for which the models are being developed) would result in improved performance.

The datasets that we assembled for each pathogen allowed us to: (i) investigate the *generalization* performance of our models to the prediction of new epitopes in proteins belonging to the specific organisms for which they were trained, by examining the predictive performance for distinct proteins that were set aside as the organism-specific *Hold-out*; (ii) investigate the effect of using only organism-specific data on predictive performance, by contrasting models developed using the *OrgSpec*, *Hybrid* and *Heterogeneous* datasets (since all pre-processing, feature development and classification models were the same for all cases, any systematic differences in performance can be attributable to the pre-selection of training data); (iii) compare the performance of organism-specific models against usual approaches in the literature. This last point was the main motivating factor for using a hold-out approach rather than cross-validation for model assessment, as it allowed us to estimate the generalization performance of all predictors on the same data rather than using reported performance values from the literature, which were obtained on distinct datasets or using different testing protocols.

#### 2.1.1 Data representation

Each dataset was set up as a fixed-width windowed representation. A sliding window of length 15 with a step size of one was run over each peptide. The choice of length 15 was based on the smallest peptide length of interest, namely 8. The rationale was to use the longest possible window such that strictly more than half the AAs covered would belong to a labelled peptide, which translates as a window of length $\ell_{\min}/2-1$, with $\ell_{\min}$ representing the shortest labelled peptide in the training sets. Based on this windowed representation the following features were calculated:

- Percent composition of the sequence in terms of each individual AA type (20 features), each dipeptide combination (400 features), each conjoint triad (Shen *et al.*, 2007; Wang *et al.*, 2017) (343 features) and each of nine AA types: Tiny, Small, Aliphatic, Aromatic, Non-Polar, Polar, Charged, Basic and Acidic (9 features).
- AA descriptors, averaged over the window: Cruciani properties, Kidera factors, Z scales, FASGAI indices, T scales, VHSE scales, ProtFP descriptors, ST Scales, BLOSUM indices and MS-WHIM scores (Osorio *et al.*, 2015) (66 features).
- Total molecular weight of the window (1 feature).
- Total number of Carbon, Hydrogen, Nitrogen, Oxygen and Sulphur atoms in the sequence (5 features).
- Entropy of the distribution of AA residues in the sequence (1 feature).

#### 2.1.2 Target pathogens used

The following organisms were used to investigate the efficiency of organism-specific training:

- *Onchocerca volvulus* (taxonomy ID: 6282), a roundworm (Nematoda) which is the causative agent of Onchocerciasis, a leading cause of blindness worldwide (World Health Organization, 2019) with over 37 million people estimated to be infected, mostly in Africa and Latin America (Basáñez *et al.*, 2006; Osei-Atweneboana *et al.*, 2007).
- Epstein-Barr Virus (taxonomy ID: 10376), a double-stranded DNA virus of the *Herpesviridae* family that is the causative agent of infectious mononucleosis and a pathogen linked to many human neoplastic diseases (Rezk *et al.*, 2018).
- Hepatitis C Virus (taxonomy ID: 11102), a positive-sense single-stranded RNA virus of the family *Flaviviridae* which causes Hepatitis C and is associated with the development of certain cancers (Ferri, 2015).

The main criterion used to select these pathogens was the availability of a large volume of validated positive and negative observations in the IEDB, to allow the use of the strict validation strategy outlined above (based on the use of a 25% hold-out set) while keeping enough data for model development. To that end we extracted the ten organism IDs with the greatest number of valid entries in the IEDB (after the filtering described in Section 2.1), and selected those that (i) had a reasonable balance between the positive and negative examples (this removed entries with heavily imbalanced class distributions, IDs 353153, 1314, 5833, 1392); and (ii) represented a pathogen of interest (this removed entries related to allergens or potential self-epitopes, IDs 9606, 9913 and 3818). The test pathogens selected based on these criteria were a multi cellular parasite, an RNA virus and a DNA virus, which allowed us to evaluate how our pathogen-specific tools perform when evaluating distinct classes of parasitic organisms. Supplementary File S1, Table S1-2 documents the dataset sizes extracted for each organism.

The results obtained for these pathogens were used to assess the performance gains of models trained with organism-specific data, and to compare their performance against existing predictors. The specific results obtained for *O.volvulus* are explored in greater detail in Section 3.1, to illustrate some particular aspects of the proposed approach. A fourth pathogen, the bacterium *Streptococcus Pyogenes*, was also investigated for the sake of completeness, although it did not fulfil the selection criteria outlined above. The specific data pre-processing, modelling and results obtained for this bacterium are described in Supplementary File S2.

### 2.2 Modelling

All training sets were used to develop Random Forest (RF) predictors (Breiman, 2001). This work used the RF implementation from R package *ranger* (R Core Team, 2020; Wright and Ziegler, 2017) version 0.12.1, under standard hyper-parameter values.Experiments with hyper-parameter tuning and feature selection did not result in relevant improvements in performance. All exploratory modelling experiments are documented in Supplementary File S5.

The final output of our predictive pipeline consists of a predicted probability for each position on each protein queried, which is converted to a binary prediction by thresholding at the level 0.5 (no tuning was performed for the threshold value). From these AA-wise predictions, arbitrary-length predicted epitopes are extracted. To reduce prediction noise, positive regions shorter than 8-AAs long were filtered out from the output of the random forest.

### 2.3 Performance assessment and comparison

Several performance indicators were calculated to provide comparability with different references in the literature, and to explore distinct aspects of the predictive behaviour of the models. More specifically, we assessed and compared model performance using the *Positive Predictive Value* (PPV), *Negative Predictive Value* (NPV), *Sensitivity* (SENS), *Accuracy* (ACC), *Area Under the ROC Curve* (AUC) (Tan *et al.*, 2005) and *Matthews Correlation Coefficient* (MCC) (Chicco and Jurman, 2020). The detailed mathematical definition and interpretation of each of these measures is provided in Supplementary File S5.

All performance values reported in the Results section refer to out-of-sample prediction, i.e. observed performance on the *Hold-out* set extracted for each individual pathogen. Since this data is not used at any point in model development, the performance values reported are considered as representing a good estimation of the generalization performance of the proposed models for these organisms.

Performance was calculated based on peptide-wise correct classifications. Following standard practice, a classification was considered as correct whenever a model predicted the right class for strictly more than half the residues in a labelled peptide. Bootstrap (Davison and Hinkley, 2013) was used to calculate standard errors of estimation for each performance measure, as well as to derive *P*-values for the comparison of mean performance between our reference implementation (trained with *OrgSpec*) and all other comparison methods (999 bootstrap resamples were used in all cases). The resulting *P*-values were corrected for multiple hypothesis testing (MHT) using the Holm correction (Holm, 1979), which provides strict control of the Family-wise error rate (FWER) for each family of hypotheses. All comparisons were done at the joint $\alpha^{⋆}=0.05$ significance level.

Five well-known B-cell epitope predictors providing easy-to-use online interfaces were used to obtain a comparison baseline: BepiPred 2.0 (Jespersen *et al.*, 2017), SVMTriP (Yao *et al.*, 2012), LBtope (Singh *et al.*, 2013), ABCpred (Saha and Raghava, 2006) and iBCE-EL (Manavalan *et al.*, 2018). These models were used to predict epitopes in the same *Hold-out* sets as our models, based on the default configurations of their respective online tools.

## 3 Results and discussion

**Organism-specific training improves performance of linear B-cell epitope prediction**

As detailed in Section 2.3, the performance of the organism-specific Random Forest models (*RF-OrgSpec*) was compared with (i) the same Random Forest model trained using heterogeneous and hybrid data, to investigate the effect on performance of the data selection strategy; and (ii) a number of well-known predictors, to provide a comparison against currently used approaches. In all cases the performance was calculated based on the hold-out set that was isolated for each pathogen, which was not used at any point in model development. Random Forests are ensemble learning methods that consist of the aggregation of several weaker decision tree (DT) models, with an output based on the combined output of the underlying DTs. Random forests present a good balance between computational cost and performance, and are robust and flexible to work with different data types and scales, which justifies their use in a variety of application domains including several epitope prediction methods (Jespersen *et al.*, 2017; Saravanan and Gautham, 2015). Preliminary comparative testing suggested Random Forests and Gradient Boosting models as having better performance than multi-layer perceptron neural networks and kNN classifiers, and RF was chosen for this work due to presenting lower computational costs in relation to Gradient Boosting.

**Fig. 2.**
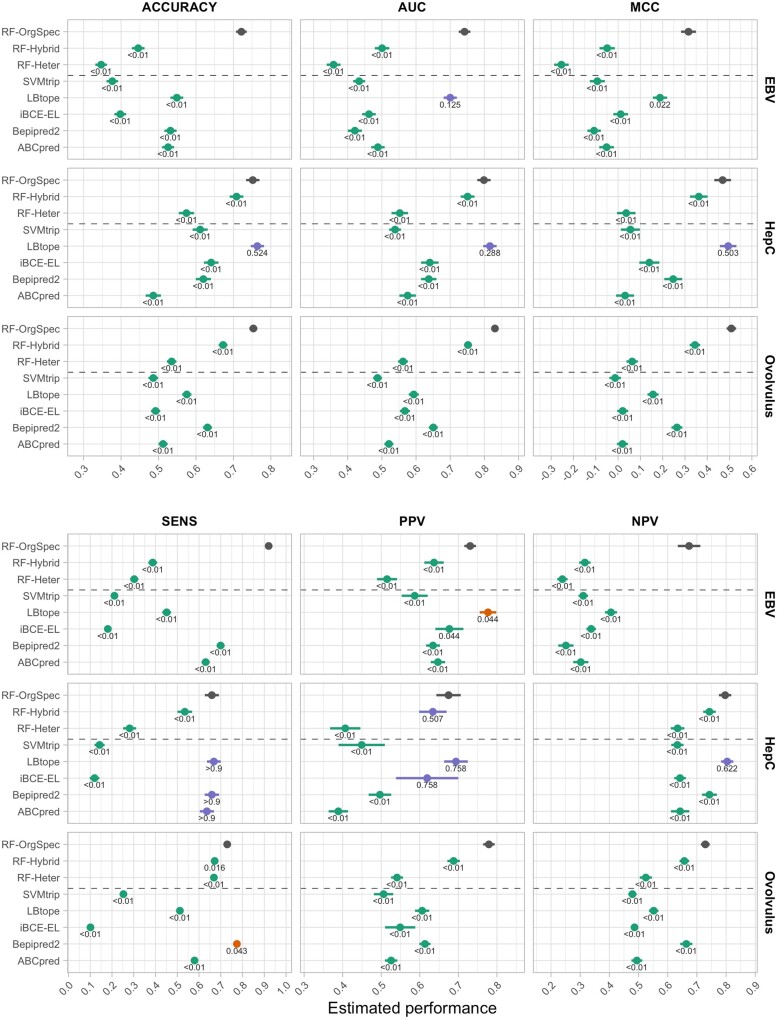
Performance estimates and standard errors of different predictors on the hold-out data of the test organisms. The values near each estimate are MHT-corrected *P*-values for the comparison of mean performance against *RF-OrgSpec*. Estimates are colour-coded for the result of significance tests at the $\alpha^{*}=0.05$ significance level (green for significantly worse than *RF-OrgSpec*, red for significantly better, blue for non-statistically significant differences). The *P*-values were truncated at < 0.01 and > 0.9 due to loss in precision of bootstrap estimates at extreme values. Raw (uncorrected) *P*-values are reported in Supplementary File S1, Table S1-4

Figure 2 summarizes the results obtained for the organisms described in Section 2.1.2. The strong positive effect of training models with organism-specific data was observed in all datasets. A clear performance ordering *RF-OrgSpec* > *RF-Hybrid* > *RF-Heter* can be observed across all pathogens, on all performance indices used. The corrected *P*-values indicate that the observed differences are in most cases statistically significant at the joint 0.05 significance level. This pattern corroborates the initial hypothesis that training models on organism-specific data yields improved predictive performance, even when compared with models that contain the same organism-specific data combined with examples from other organisms.

Additional analyses also confirm that performance gains of organism-specific prediction are observed for the pathogen for which the model is originally trained, but not when trying to predict epitopes for other organisms. Figure S3-1 in Supplementary File S3 contrasts the observed performance of OrgSpec models on the hold-out set of their specific organism with that obtained when predicting epitopes for other pathogens. These results clearly illustrate that the excellent gains in organism-specific performance (Fig. 2) come at the cost of a reduced ability to detect patterns in proteins coming from other pathogens, which further corroborates our underlying hypothesis that organism-specific training allows models to learn patterns that may be idiosyncratic to the target pathogen.

**Organism-specific models exhibit better performance than existing generalist models**

Contrasting the observed performance values of the *RF-OrgSpec* models with the selected predictors in Figure 2, it is again clear that even the standard Random Forest model used in this work (without hyper-parameter tuning or threshold adjustment) was able to significantly outperform all baseline models on most performance measures. The only predictor that presents performance values comparable to *RF-OrgSpec* is LBtope in the case of the hepatitis C virus. This can, however, be partly explained by the fact that part of the hold-out examples used to assess the performance of the models is present in the training data of LBtope (9.59% of the Hep C hold-out sequences are present in the LBtope training dataset). There is also significant presence of our hold-out Hep C examples in the training data BepiPred-2.0 (16.3%) and iBCE-EL (8.6%). Other predictors are not substantially affected, and this is not observed in the case of the other pathogens tested. Supplementary File S1,  Table S1-3 provides the performance values of all predictors on all test organisms, including performance values calculated using only the unseen sequences (not part of the training set) for the case of the hepatitis C virus.

### 3.1 Example: *O.volvulus* results

The predictions obtained for the *O.volvulus* data were selected to illustrate the organism-specific results in more detail.  Figure S3-2 in Supplementary File S3 (right panel) shows the ROC curves obtained for all predictors on the *O.volvulus* hold-out data, clearly indicating that the organism-specific model does indeed result in substantial performance gains. The *RF-OrgSpec* model presented very good robustness to different threshold values (AUC = 0.83). *RF-Hybrid*, which also included organism-specific data as part of its training set, displayed reasonably good performance as well (AUC = 0.75).

Figures S3-3 and S3-4 (Supplementary File S3) illustrate the target regions predicted by the organism-specific pipeline for the 22 hold-out proteins of the *O.volvulus* data, using the default threshold value of 0.5 ( Fig. S3-5 to S3-11 in Supplementary File S3 illustrate the corresponding results for the other pathogens tested). This illustrates not only the excellent concordance of the *RF-OrgSpec* predictions with the known labels on the hold-out proteins, but also a number of newly identified potential epitopes that may exist in those proteins. The peptides output by the *O.volvulus* model with an average probability of over 0.75 are listed in Supplementary File S1, Table S1-5.

These results show how the higher overall performance of organism-specific models, when compared with state-of-the-art predictors, can be invaluable to advance the detection and selection of diagnostic targets and vaccine candidates for infectious diseases. In particular, the higher PPV values (see Fig. 2) indicate that predicted targets have a good chance to be indeed antigenic, improving the efficiency of epitope discovery processes based on the proposed organism-specific models. This could be a consequence of idiosyncratic patterns of epitopes in different species that would be neglected by generalist predictors. For this reason, organism-specific models may be especially relevant for types of pathogens that are usually under-represented in generic epitope training data bases.

### 3.2 Discussion

The results described in this section indicate a clear improvement in performance resulting from the use of organism-specific models, when compared to generalist predictors trained on heterogeneous, or even hybrid, data. While an in-depth exploration of the underlying causes of these differences in performance is outside the scope of this work, there are some potential, non-mutually exclusive hypotheses that could be raised.

An examination of the relevance of distinct features and feature groups is provided in Supplementary File S6. There are interesting general insights that can be derived from that exploration in terms of which feature groups contribute the most to the predictive ability of both OrgSpec and Heterogeneous models (Supplementary File S6,  Fig. S6-1 to S6-3), such as the disproportionately large prevalence of *AA descriptors*-type features among the most relevant, of the apparent irrelevance of dipeptide frequencies or Conjoint Triads for the linear B-cell epitope problem as modelled here. However, it is potentially more valuable in the context of this particular work to focus on features that appear more consistently as relevant for OrgSpec models than Heterogeneous ones. As suggested in Supplementary File S6 (Supplementary Fig. S6-4–6), feature BLOSUM1 (Georgiev, 2009) is clearly one that stands out in terms of being very relevant in general, and particularly so for the organism-specific models. This feature is very strongly correlated with hydrophobicity, with $r^{2}=0.94$ according to (Georgiev, 2009). For the windowed data representation used in this work, it measures the average hydrophobicity of the 15-AA neighbourhood of a given position on the protein. Hydrophobicity/hydrophilicity are directly related to epitope accessibility in the protein structure. Hydrophilic polar regions are usually observed in the protein surface, been constantly exposed to antibodies, whereas hydrophobic regions often interact either with each other in the protein core or with other cellular components, and are not readily accessible to the serologic immune response (Hopp and Woods, 1981). Other features that seem to appear consistently amongst the most relevant ones (although not as prominently as BLOSUM1), e.g. ProtFP1, Z1, VHSE8 and F5 (see Supplementary File S6,  Fig. S6-6) are composite scales based on algebraic transformations of underlying physicochemical properties, and lack the same direct interpretability as BLOSUM1, which prevents the derivation of biochemical hypotheses.

Another aspect that may suggest an explanation for the increased performance of organism-specific models is a possible difference in the spatial distribution of epitopes in the feature space, conditional on the pathogen. To explore the neighbourhood structure of the data, we have employed t-SNE projections (Van der Maaten and Hinton, 2008) to investigate whether data coming from distinct pathogens present different clustering or neighbourhood structures in terms of positive/negative observations.

**Fig. 3.**
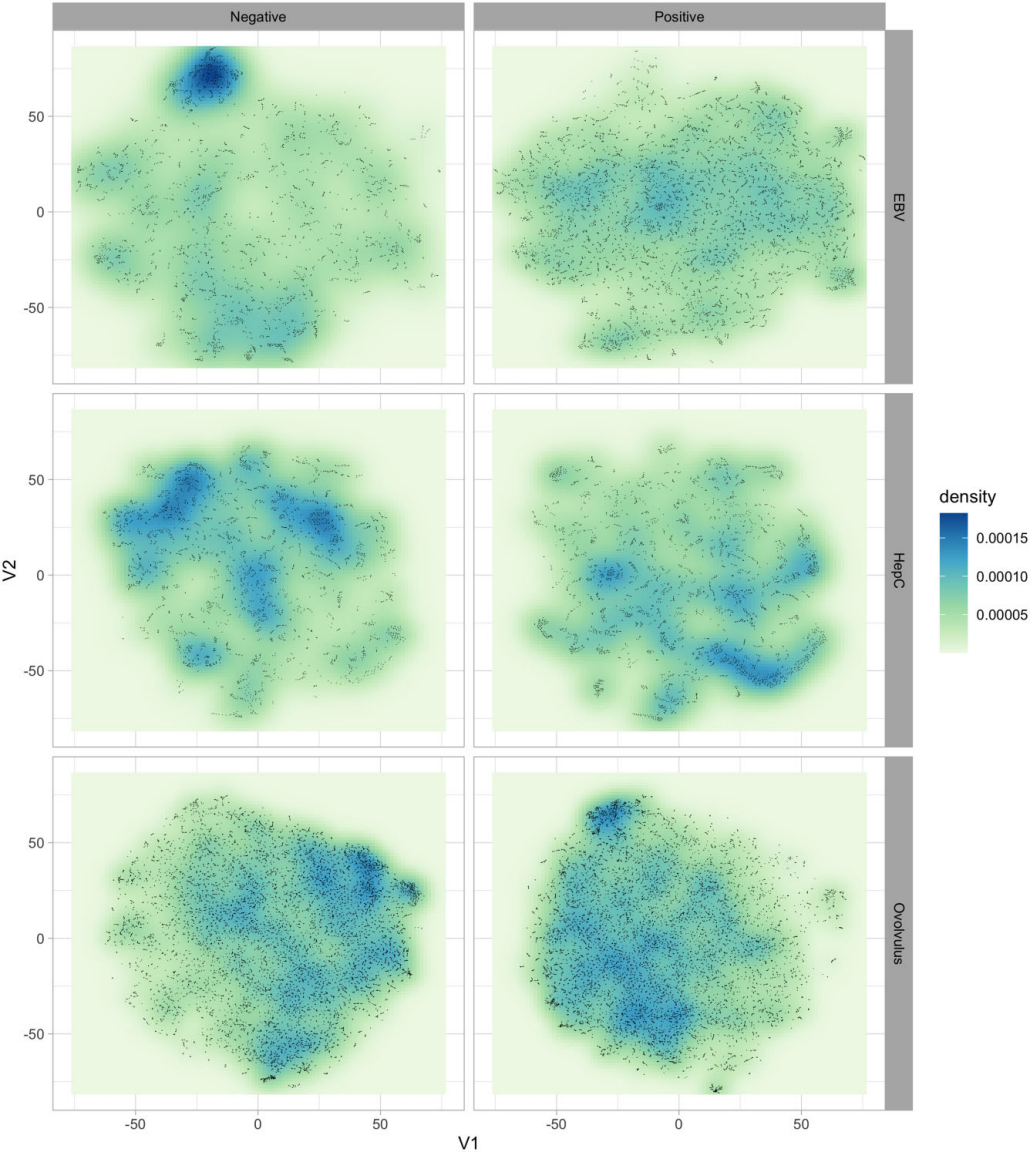
Estimated probability density of epitope and non-epitope observations in the t-SNE projection. Notice the clear distinct regions of high density of positive/negative observations, which occupy different portions of the feature space. This figure clearly illustrates how epitopes (*positive* observations) of different pathogens tend to occur in very distinct regions of the space of features. More importantly, regions that present a high density of positive examples for one pathogen can simultaneously have high numbers of negative observations for another—see, e.g. how the top-left portion of the negative examples of EBV and HepC coincide with a corresponding high-density regions of positive *O.volvulus* points. Models trained on combined (heterogeneous) data would not be able to explore these patterns, and would likely fail to detect promising regions, which may explain the increased performance of the organism-specific models when compared against generalist ones trained on heterogeneous data

Figure 3 illustrates the estimated density of observations on the 2D t-SNE projection of the data, stratified by pathogen and class. The V1-V2 coordinates are consistent across the different panels, and the figure clearly shows how the density of positive and negative examples not only varies depending on the pathogen, but also how regions with a high density of positive examples for one organism can simultaneously contain high densities of negative examples for others. This type of pattern can help explain the success of organism-specific training from a data mining perspective (albeit not necessarily from a biological one): generalist models trained on heterogeneous data would not be able to pick up these organism-specific patterns, as they would appear as having a more mixed combination of positive and negative examples if the data from multiple pathogens were combined into a single training set. This could in effect prevent those models from detecting regions of the feature space that were potentially rich in epitopes of a specific pathogen, resulting in decreased predictive performance.

## 4 Conclusions

In this article, we investigated the use of organism-specific data for improving the performance of linear B-cell epitope prediction. Organism-specific Random Forest models developed for three distinct pathogens (Epstein-Barr virus, Hepatitis C virus and the roundworm *O.volvulus*) yielded significant performance gains when compared with similar models trained using heterogeneous and hybrid datasets, across several relevant performance indicators. These results suggest that pre-selecting the most relevant data and training bespoke models for specific pathogens is preferable to the common strategy of increasing and diversifying the training set.

Performance comparisons also indicate that this organism-specific modelling strategy is able to provide results that are at least as good as, and in several cases better than, several common predictors from the literature, despite the fact that (i) the predictors trained in this study were relatively simple proof-of-concept models, without specific refinements; and (ii) only basic features, calculated from the AA sequence alone, were employed, without any sophisticated feature engineering performed. We expect that further refinements to organism-specific predictors, such as model improvements or the use of more informative features, may result in even higher predictive performance. While these results do not obviate the utility of generalist predictors—which are still very relevant in the investigation of pathogens for which little or no specific data is available—they certainly suggest a powerful and easily generalizable new approach for researchers working with relatively data-abundant organisms.

## Supplementary Material

btab536_Supplementary_Data
