# Supplementary material for: Organism-specific training improves performance of linear B-cell epitope prediction
Source: Bioinformatics. 2021 Jul 21;37(24):4826–34. doi: 10.1093/bioinformatics/btab536 (PMC8665745; doi:10.1093/bioinformatics/btab536)
Supplement: btab536_Supplementary_Data [file btab536_supplementary_data.zip › 00_2_Supplementary_File_1.pdf]

Supplementary File 1: Supplementary Tables

July 1, 2021

Table S1 - 1: Epitope prediction tools in the literature and the composition of their training data sets. PK = prokaryotes; VI = virus; FG = fungi; PR = protozoan; HM = human; OE = other eukaryotes. Details on the source databases used can be found on the works cited in the *Methods* column.

**Method**	**Year**	**Training dataset**	
		**Sources**	**Composition**
Antigenic (Kolaskar and Tongaonkar, 1990)	1990	-	-
PREDITOP (Pellequer and Westhof, 1993)	1993	-	-
People (Alix, 1999)	1999	-	-
Bepitope (Odorico and Pellequer, 2003)	2003	-	-
Bcepred (Saha and Raghava, 2004)	2004	-	-
Söllner/Mayer (Söllner and Mayer, 2006)	2006	BCIPEP, FIMM	PK+VI+FG+PR+OE
ABCpred (Saha and Raghava, 2006)	2006	BCIPEP	PK+VI+FG+PR+OE
Chen (Chen *et al.*, 2007)	2007	BCIPEP	PK+VI+FG+PR+OE
BCpred (EL-Manzalawy *et al.*, 2008)	2008	BCIPEP	PK+VI+FG+PR+OE
FBCpred (EL-Manzalawy *et al.*, 2008)	2008	BCIPEP	PK+VI+FG+PR+OE
LEPD (Chang *et al.*, 2008)	2008	Antijen, Pellequer	PK+VI+PR+HM+OE
Epitopia (Rubinstein *et al.*, 2009)	2009	BCIPEP	PK+VI+FG+PR+OE
COBEpro (Sweredoski and Baldi, 2009)	2009	BCIPEP, HIV	PK+VI+FG+PR+OE
BayesB (Wee *et al.*, 2010)	2010	BCIPEP	PK+VI+FG+PR+OE
LEPS (Wang *et al.*, 2011a)	2011	BCIPEP	PK+VI+FG+PR+OE
BEOracle (Wang *et al.*, 2011b)	2011	BCIPEP, IEDB, Antijen	PK+VI+FG+HM+OE
SVMTriP (Yao *et al.*, 2012)	2012	IEDB	PK+VI+PR+HM+OE
BEST (Gao *et al.*, 2012)	2012	BCIPEP, SWISS-PROT	PK+VI+OE
LBtope (Singh *et al.*, 2013)	2013	IEDB	PK+VI+HM+PR+OE
BeePro (Lin *et al.*, 2013)	2013	Mix of datasets	PK+VI+FG+PR+HM+OE
EPMLR (Lian *et al.*, 2014)	2014	BCIPEP, IEDB, Antijen	PK+VI+FG+HM+OE
DMN-LBE (Lian *et al.*, 2015)	2015	IEDB	PK+VI+HM+PR+OE
LBEEP (Saravanan and Gautham, 2015)	2015	IEDB	PK+VI+HM+PR+OE
APCpred (Shen *et al.*, 2015)	2015	BCIPEP	PK+VI+FG+PR+OE
Bepipred 2.0 (Jespersen *et al.*, 2017)	2017	PDB	PK+VI+OE
DRREP (Sher *et al.*, 2017)	2017	BCIPEP	PK+VI+FG+PR+OE
iBCE-EL (Manavalan *et al.*, 2018)	2018	IEDB	PK+VI+HM+PR+OE
EpiDope (Collatz *et al.*, 2020)	2020	IEDB	PK+VI+HM+PR+OE

Table S1 - 2: Number of positive/negative examples in each dataset. Hold-out sets always contain only target organism proteins that were not seen during model training.

		Orgspec	Heter.	Hybrid	Hold-out
O. volvulus	Positive	2441	2634	5075	832
	Negative	2378	2922	5300	777
Epstein-Barr	Positive	1746	1981	3727	625
	Negative	811	1864	2675	315
Hep. C virus	Positive	919	1926	2845	218
	Negative	783	1975	2758	358

Table S1 - 3: Estimates and standard errors of performance for all models tested. Of the three test organisms used, only the Hepatitis C virus had a significant presence of hold-out observations as part of the training sets of some of the benchmark predictors (16.3% for Bepipred2, 8.6% for iBCE-EL and 9.59% for LBtope). Values in parentheses indicate the performance variation (up or down) observed when only sequences not present in the training set were used for validation. Notice the substantial drop in performance for LBtope in this case. For the other organisms this type of *leakage* was minimal and did not affect the estimates, so the full hold-out set was used for assessment and a single estimate is presented.

	Epstein-Barr Virus					
	ACC	AUC	MCC	PPV	NPV	SENS
RF-OrgSpec	0.72 ± 0.01	0.74 ± 0.02	0.32 ± 0.03	0.73 ± 0.02	0.67 ± 0.04	0.92 ± 0.01
RF-Hybrid	0.45 ± 0.02	0.5 ± 0.02	-0.05 ± 0.03	0.64 ± 0.02	0.32 ± 0.02	0.39 ± 0.02
RF-Heter	0.35 ± 0.02	0.36 ± 0.02	-0.25 ± 0.03	0.51 ± 0.03	0.24 ± 0.02	0.3 ± 0.02
ABCpred	0.53 ± 0.02	0.49 ± 0.02	-0.05 ± 0.03	0.65 ± 0.02	0.3 ± 0.03	0.63 ± 0.02
Bepipred2	0.53 ± 0.02	0.42 ± 0.02	-0.11 ± 0.03	0.63 ± 0.02	0.25 ± 0.03	0.7 ± 0.02
iBCE-EL	0.4 ± 0.02	0.46 ± 0.02	0.01 ± 0.03	0.68 ± 0.04	0.34 ± 0.02	0.18 ± 0.02
LBtope	0.55 ± 0.02	0.7 ± 0.02	0.19 ± 0.03	0.78 ± 0.02	0.41 ± 0.02	0.45 ± 0.02
SVMtrip	0.38 ± 0.02	0.43 ± 0.02	-0.09 ± 0.03	0.59 ± 0.03	0.31 ± 0.02	0.21 ± 0.02

	O. volvulus					
	ACC	AUC	MCC	PPV	NPV	SENS
RF-OrgSpec	0.75 ± 0.01	0.83 ± 0.01	0.51 ± 0.02	0.78 ± 0.01	0.73 ± 0.02	0.73 ± 0.02
RF-Hybrid	0.67 ± 0.01	0.75 ± 0.01	0.34 ± 0.02	0.69 ± 0.02	0.66 ± 0.02	0.67 ± 0.02
RF-Heter	0.54 ± 0.01	0.56 ± 0.01	0.06 ± 0.03	0.54 ± 0.02	0.52 ± 0.02	0.67 ± 0.02
ABCpred	0.51 ± 0.01	0.52 ± 0.01	0.02 ± 0.02	0.53 ± 0.02	0.49 ± 0.02	0.58 ± 0.02
Bepipred2	0.63 ± 0.01	0.65 ± 0.01	0.26 ± 0.02	0.61 ± 0.01	0.66 ± 0.02	0.77 ± 0.01
iBCE-EL	0.49 ± 0.01	0.57 ± 0.01	0.02 ± 0.03	0.55 ± 0.04	0.49 ± 0.01	0.1 ± 0.01
LBtope	0.58 ± 0.01	0.59 ± 0.01	0.16 ± 0.02	0.61 ± 0.02	0.55 ± 0.02	0.51 ± 0.02
SVMtrip	0.49 ± 0.01	0.49 ± 0.01	-0.01 ± 0.02	0.51 ± 0.02	0.48 ± 0.01	0.25 ± 0.01

	Hepatitis C Virus					
	ACC	AUC	MCC	PPV	NPV	SENS
RF-OrgSpec	0.75 ± 0.02	0.8 ± 0.02	0.47 ± 0.04	0.67 ± 0.03	0.8 ± 0.02	0.66 ± 0.03 $(0.01 \uparrow)$
RF-Hybrid	0.71 ± 0.02	0.75 ± 0.02	0.36 ± 0.04	0.63 ± 0.04	0.74 ± 0.02 $(0.01 \uparrow)$	0.53 ± 0.03 $(0.01 \uparrow)$
RF-Heter	0.57 ± 0.02	0.55 ± 0.02	0.04 ± 0.04	0.41 ± 0.04	0.63 ± 0.02	0.28 ± 0.03
ABCpred	0.49 ± 0.02	0.58 ± 0.02	0.03 ± 0.04	0.39 ± 0.03	0.64 ± 0.03	0.64 ± 0.03
Bepipred2	0.62 ± 0.02 $(0.02 \downarrow)$	0.64 ± 0.02 $(0.04 \downarrow)$	0.25 ± 0.04 $(0.03 \downarrow)$	0.5 ± 0.03 $(0.01 \uparrow)$	0.74 ± 0.03 $(0.03 \downarrow)$	0.66 ± 0.03 $(0.03 \downarrow)$
iBCE-EL	0.64 ± 0.02 $(0.02 \downarrow)$	0.64 ± 0.02	0.14 ± 0.04 $(0.02 \uparrow)$	0.62 ± 0.08 $(0.10 \uparrow)$	0.64 ± 0.02 $(0.02 \downarrow)$	0.12 ± 0.02 $(0.02 \downarrow)$
LBtope	0.76 ± 0.02	0.82 ± 0.02 $(0.11 \downarrow)$	0.49 ± 0.04 $(0.27 \downarrow)$	0.69 ± 0.03 $(0.38 \downarrow)$	0.8 ± 0.02 $(0.08 \uparrow)$	0.67 ± 0.03 $(0.25 \downarrow)$
SVMtrip	0.61 ± 0.02	0.54 ± 0.02 $(0.01 \downarrow)$	0.06 ± 0.04 $(0.02 \downarrow)$	0.45 ± 0.06 $(0.03 \downarrow)$	0.63 ± 0.02	0.14 ± 0.02 $(0.01 \downarrow)$

Table S1 - 4: Raw (uncorrected) p-values for all comparisons of organism-specific models with other predictors, shown in Figure 2. The smallest detectable p-value by the bootstrap approach used was 0.001. For the hypotheses tested in this work this level of precision was sufficient.

	Epstein-Barr Virus					
	SENS	ACC	PPV	NPV	MCC	AUC
vs. RF-Heter	≤0.001	≤0.001	≤0.001	≤0.001	≤0.001	≤0.001
vs. RF-Hybrid	≤0.001	≤0.001	≤0.001	≤0.001	≤0.001	≤0.001
vs. ABCpred	≤0.001	≤0.001	≤0.001	≤0.001	≤0.001	≤0.001
vs. Bepipred2	≤0.001	≤0.001	≤0.001	≤0.001	≤0.001	≤0.001
vs. iBCE-EL	≤0.001	≤0.001	0.036	≤0.001	≤0.001	≤0.001
vs. LBtope	≤0.001	≤0.001	0.022	≤0.001	0.022	0.125
vs. SVMtrip	≤0.001	≤0.001	≤0.001	≤0.001	≤0.001	≤0.001

	O. Volvulus					
	SENS	ACC	PPV	NPV	MCC	AUC
vs. RF-Heter	≤0.001	≤0.001	≤0.001	≤0.001	≤0.001	≤0.001
vs. RF-Hybrid	0.008	≤0.001	≤0.001	≤0.001	≤0.001	≤0.001
vs. ABCpred	≤0.001	≤0.001	≤0.001	≤0.001	≤0.001	≤0.001
vs. Bepipred2	0.043	≤0.001	≤0.001	≤0.001	≤0.001	≤0.001
vs. iBCE-EL	≤0.001	≤0.001	≤0.001	≤0.001	≤0.001	≤0.001
vs. LBtope	≤0.001	≤0.001	≤0.001	≤0.001	≤0.001	≤0.001
vs. SVMtrip	≤0.001	≤0.001	≤0.001	≤0.001	≤0.001	≤0.001

	Hepatitis C Virus					
	SENS	ACC	PPV	NPV	MCC	AUC
vs. RF-Heter	≤0.001	≤0.001	≤0.001	≤0.001	≤0.001	≤0.001
vs. RF-Hybrid	≤0.001	≤0.001	0.169	≤0.001	≤0.001	≤0.001
vs. ABCpred	0.629	≤0.001	≤0.001	≤0.001	≤0.001	≤0.001
vs. Bepipred2	1.000	≤0.001	≤0.001	≤0.001	≤0.001	≤0.001
vs. iBCE-EL	≤0.001	≤0.001	0.379	≤0.001	≤0.001	≤0.001
vs. LBtope	0.867	0.524	0.491	0.622	0.503	0.288
vs. SVMtrip	≤0.001	≤0.001	≤0.001	≤0.001	≤0.001	≤0.001

Table S1 - 5: Predicted target regions in proteins of the *O. volvulus* hold-out set with average predicted probabilities above 0.75. Column *Matches* indicate IEDB epitope IDs for which the sequence identity returned a match of over 80%. Notice that (i) only predictions for proteins in the **hold-out** set are provided here, since the objective of this paper is to demonstrate the methodology rather than to provide an in-depth investigation of *O. volvulus* epitopes; and (ii) two candidate epitopes have no match on IEDB (Matches = **N/A**), and may represent new targets for experimental investigation

**Protein**	**Start**	**End**	**Length**	**Prob.**	**Sequence**	**Matches**
A0A044UVG8	845	882	38	0.86	ERSVSQCKTSDSRNRSSSRRNSPEYDISQI TEQESEKS	852235 (100%); 855900 (100%)
AAD00842.1	1	71	71	0.83	NADEKNENYSLNIKKMDASKEIDSNNKFKN PIENEQEDCLSNDEELQKDSRGVIQTDEEG DKCESPKQEVN	851260 (100%); 856295 (100%); 853774 (100%); 854736 (100%); 854918 (93.333%)
A0A044V9S3	43	85	43	0.81	DVEVQLWEEDTGDPDDLLNSTRSDEKGAFE VYGEDKEVTAIEP	854970 (100%); 851810 (100%); 852047 (100%); 857301 (100%); 857276 (100%); 851449 (87.5%); 854446 (85.714%)
A0A044SS43	15	90	76	0.80	LIIVKNADEKNENYSLNIKKMDASKEIDSN NKFKNPIENEQEDCRSNDEELQKDSRGVIQ TDEEGDKCESPKQEVN	854918 (100%); 851260 (100%); 856295 (100%); 853774 (100%); 854736 (100%); 856311 (85.714%)
AAF64251.1	120	149	30	0.80	FVHHGLSGNAIESLFNEVDKNGDGYLTSIE	854055 (100%); 853779 (100%); 852831 (93.333%)
A0A2K6VRB1	299	349	51	0.80	GPPGPPGNPGQDGRQGPQGDPGKDGLPGKP GIDGTTGPQGERGPSGSCDHC	855140 (100%); 855121 (100%); 855119 (100%); 851603 (100%); 855456 (100%); 853564 (90%)
AAF64251.1	376	394	19	0.80	FDILQEIDSDGDGYINNSE	854291 (100%); 853775 (100%); 851576 (100%)
A0A044SAZ1	425	442	18	0.80	AYQIEKDNPNFNANNDLI	855469 (100%); 855469 (100%); 854673 (100%); 855208 (100%); 945036 (83.333%); 945545 (83.333%)
A0A044SS43	875	901	27	0.80	KWISSEEDSISELSDNYQSEESNSSGD	856192 (100%)
A0A044SAZ1	577	601	25	0.79	IGAFSVDGAEEADQKNNNHEFDLIN	851740 (100%)

Table S1 - 5 – continued from previous page

**Protein**	**Start**	**End**	**Length**	**Prob.**	**Sequence**	**Matches**
A0A044UVG8	829	841	13	0.79	SKSRSESLVVSRQ	**N/A**
A0A044SS43	945	970	26	0.79	PSLTVDNDGDEEEEEDEEEIEERLKN	853848 (100%); 856088 (83.333%)
A0A044SS43	743	766	24	0.78	KRIPISMENNQENDGFDGQKAIKM	853390 (100%); 854905 (100%); 854712 (100%)
A0A044SS43	977	1087	111	0.78	GRKIESSDEFEPDEEEEEVEDEEEEVEDEE EEENEETSTIDDEKQRQSDEEEGLEEKERK IVKVENDMKDMKEITFSSTKTSFIDDDKKQ TEDIDDVCPSTSNDVKLTDET	856569 (100%); 855321 (100%); 852138 (100%); 856604 (100%); 851924 (100%)
AAF64251.1	516	542	27	0.78	SQRPHDSSKSNAQDEEQSEKTEEVREQ	856224 (100%)
A0A044SS43	779	819	41	0.77	RLNDLNVDEMTESDTDEYQASDSASVSDPS DDTDEYLPSDA	856056 (100%)
A0A044RF80	1485	1501	17	0.77	ACPECWQTEEPNEISAG	854139 (100%); 851875 (100%)
A0A044SS43	636	659	24	0.77	TVRDAKVENSESSEESGEDDGERK	851949 (100%); 854897 (100%); 852938 (83.333%)
A0A044SS43	423	485	63	0.77	QQNKKQMEIENAKQLKILDRNDDQDDSEDN DENEKDENDADDFILAETSTSSDDDFMPLS ELK	853828 (100%); 852292 (100%); 853331 (100%); 854720 (100%); 853875 (100%); 851706 (100%)
A0A044UVG8	1	141	141	0.77	MQKNDGNEMICIEENITQKNPTDELEFITE EGYNIGANDNSEIAHEEPDFSEYGTLATKD CGEIAVDETCLHSGIREGENFGGEKFDVIE QDTKENQSSDGNSTGNVITELANQNASELS QNKVEEAVHDSSETAETRSIY	853160 (100%); 851567 (100%); 854638 (100%); 852144 (100%); 853634 (100%); 854011 (100%); 854765 (100%); 853789 (100%); 855619 (100%)
A0A044UVG8	1400	1458	59	0.77	GLKQHIDDDGDIKQQQQKQLQNAMSNYNRE QLESDASNNLILELQQRIGGSNENALALL	854721 (100%); 855565 (100%)
A0A044UVG8	1059	1094	36	0.77	MVNYATSESEAEDNITVESKSSAEQLSENY NKGVSS	855021 (100%); 856367 (100%)
AAF64254.1	328	345	18	0.77	ATRLGQSDFDDLENQPVV	854085 (100%)
A0A044UVG8	661	688	28	0.77	VGNSYESNIENEILGKHDDKDEHILVSH	854757 (100%); 856403 (100%)
A0A044TU88	308	354	47	0.76	NKLSLQKNERDKEKIEGVEKKGESKEEKKE NKEAKDYKEGDKEKEKE	852155 (100%); 851711 (100%); 853615 (100%)
A0A044SS43	915	929	15	0.76	RASSSDAEDSSHSVQ	**N/A**
A0A044QWA5	421	457	37	0.75	NQSFELQEETTNDELIDFNSLRMIDNNADP IWTFQEV	854962 (100%); 851542 (100%); 851715 (100%); 855420 (100%)

Table S1 - 5 – continued from previous page

Protein	Start	End	Length	Prob.	Sequence	Matches
AAF64251.1	239	294	56	0.75	MKNADKNDDGHIDKKEAQKIAFEQEGISAG DVNEMIMSVDDNNDGELNAPEFADFE	856870 (100%); 853605 (100%); 852659 (100%); 851234 (100%)

Supplementary References 1

Alix, A. J. (1999). Predictive estimation of protein linear epitopes by using the program people. *Vaccine*, **18**(3-4), 311–314.

Chang, H.-T., Liu, C.-H., and Pai, T.-W. (2008). Estimation and extraction of b-cell linear epitopes predicted by mathematical morphology approaches. *Journal of Molecular Recognition*, **21**(6), 431–441.

Chen, J., Liu, H., Yang, J., and Chou, K.-C. (2007). Prediction of linear b-cell epitopes using amino acid pair antigenicity scale. *Amino acids*, **33**(3), 423–428.

Collatz, M., Mock, F., Barth, E., Hölzer, M., Sachse, K., and Marz, M. (2020). EpiDope: a deep neural network for linear b-cell epitope prediction. *Bioinformatics*.

EL-Manzalawy, Y., Dobbs, D., and Honavar, V. (2008). Predicting linear b-cell epitopes using string kernels. *Journal of Molecular Recognition: An Interdisciplinary Journal*, **21**(4), 243–255.

Gao, J., Faraggi, E., Zhou, Y., Ruan, J., and Kurgan, L. (2012). Best: Improved prediction of b-cell epitopes from antigen sequences. *Plos One*, **7**(6), e40104.

Jespersen, M. C., Peters, B., Nielsen, M., and Marcatili, P. (2017). Bepipred-2.0: improving sequence-based b-cell epitope prediction using conformational epitopes. *Nucleic acids research*, **45**(W1), W24–W29.

Kolaskar, A. and Tongaonkar, P. C. (1990). A semi-empirical method for prediction of antigenic determinants on protein antigens. *FEBS letters*, **276**(1-2), 172–174.

Lian, Y., Ge, M., and Pan, X.-M. (2014). EPMLR: sequence-based linear b-cell epitope prediction method using multiple linear regression. *BMC Bioinformatics*, **15**(1).

Lian, Y., Huang, Z.-C., Ge, M., and Pan, X.-M. (2015). An improved method for predicting linear b-cell epitope using deep maxout networks. *Biomedical and Environmental Sciences*, **28**(6), 460–463.

Lin, S. Y.-H., Cheng, C.-W., and Su, E. C.-Y. (2013). Prediction of b-cell epitopes using evolutionary information and propensity scales. In *BMC bioinformatics*, volume 14, page S10. Springer.

Manavalan, B., Govindaraj, R. G., Shin, T. H., Kim, M. O., and Lee, G. (2018). iBCE-EL: A new ensemble learning framework for improved linear b-cell epitope prediction. *Frontiers in Immunology*, **9**.

Odorico, M. and Pellequer, J.-L. (2003). Bepitope: predicting the location of continuous epitopes and patterns in proteins. *Journal of Molecular Recognition*, **16**(1), 20–22.

Pellequer, J. and Westhof, E. (1993). Preditop: a program for antigenicity prediction. *Journal of molecular graphics*, **11**(3), 204–210.

Rubinstein, N. D., Mayrose, I., and Pupko, T. (2009). A machine-learning approach for predicting b-cell epitopes. *Molecular Immunology*, **46**(5), 840–847.

Saha, S. and Raghava, G. P. S. (2004). Bcepred: prediction of continuous b-cell epitopes in antigenic sequences using physico-chemical properties. In *International Conference on Artificial Immune Systems*, pages 197–204. Springer.

Saha, S. and Raghava, G. P. S. (2006). Prediction of continuous b-cell epitopes in an antigen using recurrent neural network. *Proteins: Structure, Function, and Bioinformatics*, **65**(1), 40–48.

Saravanan, V. and Gautham, N. (2015). Harnessing computational biology for exact linear b-cell epitope prediction: a novel amino acid composition-based feature descriptor. *Omics: a journal of integrative biology*, **19**(10), 648–658.

Shen, W., Cao, Y., Cha, L., Zhang, X., Ying, X., Zhang, W., Ge, K., Li, W., and Zhong, L. (2015). Predicting linear b-cell epitopes using amino acid anchoring pair composition. *BioData mining*, **8**(1), 1–12.

Sher, G., Zhi, D., and Zhang, S. (2017). DRREP: deep ridge regressed epitope predictor. *BMC Genomics*, **18**(S6).

Singh, H., Ansari, H. R., and Raghava, G. P. (2013). Improved method for linear b-cell epitope prediction using antigen's primary sequence. *PloS one*, **8**(5).

Söllner, J. and Mayer, B. (2006). Machine learning approaches for prediction of linear b-cell epitopes on proteins. *Journal of Molecular Recognition: An Interdisciplinary Journal*, **19**(3), 200–208.

Sweredoski, M. J. and Baldi, P. (2009). Cobepro: a novel system for predicting continuous b-cell epitopes. *Protein Engineering, Design & Selection*, **22**(3), 113–120.

Wang, H.-W., Lin, Y.-C., Pai, T.-W., and Chang, H.-T. (2011a). Prediction of b-cell linear epitopes with a combination of support vector machine classification and amino acid propensity identification. *Journal of Biomedicine and Biotechnology*, **2011**, 1–12.

Wang, Y., Wu, W., Negre, N. N., White, K. P., Cheng, L., and Shah, P. K. (2011b). Determinants of antigenicity and specificity in immune response for protein sequences. *BMC Bioinformatics*, **12**, 251.

Wee, L. J., Simarmata, D., Kam, Y.-W., Ng, L. F., and Tong, J. C. (2010). Svm-based prediction of linear b-cell epitopes using bayes feature extraction. In *BMC genomics*, volume 11, page S21. Springer.

Yao, B., Zhang, L., Liang, S., and Zhang, C. (2012). Svmtrip: a method to predict antigenic epitopes using support vector machine to integrate tri-peptide similarity and propensity. *PloS one*, **7**(9), e45152.
