# Supplementary material for: Organism-specific training improves performance of linear B-cell epitope prediction
Source: Bioinformatics. 2021 Jul 21;37(24):4826–34. doi: 10.1093/bioinformatics/btab536 (PMC8665745; doi:10.1093/bioinformatics/btab536)
Supplement: btab536_Supplementary_Data [file btab536_supplementary_data.zip › 00_2_Supplementary_File_2.pdf]

Supplementary File 2

July 1, 2021

Organism-specific model for *S. pyogenes*

Streptococcus pyogenes (taxonomy ID: 1314) is a gram-positive bacteria that is associated not only with a large number of throat and skin infections in humans, but also with invasive infections estimated to kill hundreds of thousands of people each year (O'Loughlin *et al.*, 2007). Although this pathogen is well-represented in IEDB, the vast majority of available peptides represent negative observations (6829 / 7108 entries, 96.1%). This severe imbalance in the class distribution disqualified this organism from the list of test pathogens selected in Section 2.1.2 of the paper.

While strategies to deal with heavily data-imbalanced pathogens are beyond the scope of this paper, we decided to apply a basic sub-sampling strategy to reduce the imbalance and repeat the experiment for this organism, in order to verify the performance of organism-specific models trained for a bacterial pathogen. The sub-sampling approach used in this case consisted of: (i) remove all proteins containing only negative examples from the organism-specific data; (ii) manually removing proteins with very high ratios of negative-to-positive observations, as long as the number of positive observations was not too large. The full code to reproduce this subsampling is available in `https://github.com/fcampelo/OrgSpec-paper`. The process yielded a dataset that, while still imbalanced (629 / 831, 75.4% negative observations) is far less extremely so than the original one, with minimal loss of positive examples.

After this pre-filtering of the data, the organism-specific model for *S. pyogenes* was generated following the same procedures used for the other pathogens, as described in Section 2 of the paper. Figure S2 - 1 illustrates the performance of the *OrgSpec* model in comparison with *Heter*, *Hybrid* and the five benchmark predictors.

The results obtained for *S. pyogenes* seem to generally agree with those observed for the other pathogens investigated. The organism-specific model presents a very competitive performance across all metrics, yielding results that are considerably better than the model trained with heterogeneous data, as well as than iBCE-EL and Bepipred2. The lower statistical power of the comparisons in the case of *S. pyogenes* (which is a consequence of the considerably smaller size of the hold-out set, composed of only 159 negative and 22 positive

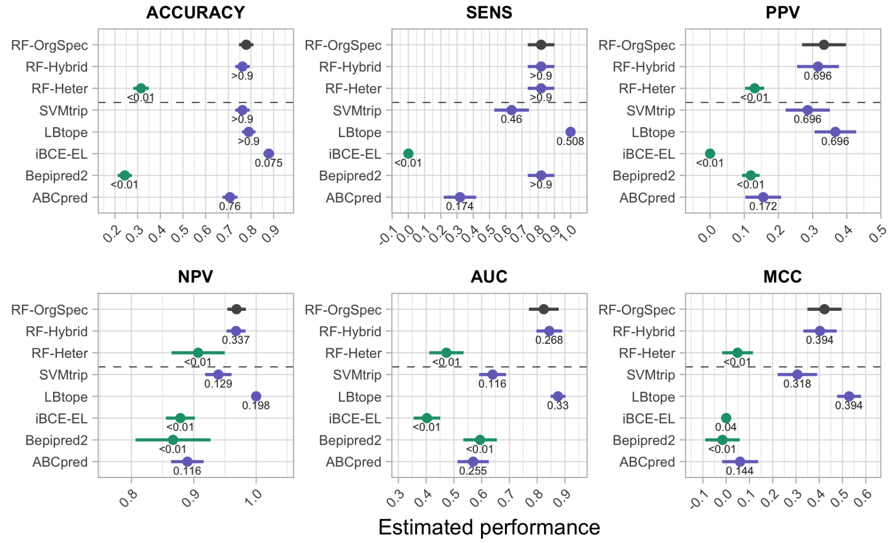

Figure S2 - 1: Performance estimates and standard errors of different predictors on the hold-out data of *S. pyogenes*, together with MHT-corrected p-values for the comparison of mean performance of each predictor against *RF-OrgSpec*. Estimates are colour-coded for the result of significance tests at the $\alpha^* = 0.05$ significance level (green for significantly worse than *RF-OrgSpec*, red for significantly better, blue for non-statistically significant differences). Raw (uncorrected) p-values are reported in Table S2 - 1.

examples[1]) resulted in several comparisons that were not significant at the joint 0.05 significance level despite considerable differences in the point estimates. While the results obtained for the other organisms would suggest that the differences observed may indeed be due to actual positive effects of organism-specific training on the predictive performance, it remains to be further validated on other bacterial organisms after better class-imbalanced classification strategies are deployed as part of the organism-specific predictive pipeline. Regardless, it is clear from Figure S2 - 1 that organism-specific training did yield a model that was clearly non-inferior to the generalist ones tested, and in several cases significantly better.

One aspect of the *S. pyogenes* results that differs from the other organisms is the observation that the *Hybrid* and *OrgSpec* models performed similarly for this case, whereas for the others the purely organism-specific training generally outperformed the models developed with hybrid data. Although the reasons for this difference remain unclear (it may be due to a compound effect of the specific composition of the heterogeneous data set for the *S. pyogenes* case study

[1]See Supplementary File 1, Table S1 - 2 for the corresponding sample sizes for the other pathogens tested

Table S2 - 1: Raw (uncorrected) p-values for all comparisons shown in Figure S2 - 1. The smallest detectable p-value by the bootstrap approach used was 0.001. For the hypotheses tested in this work, this level of precision was sufficient.

***OrgSpec* vs.**	**SENS**	**ACC**	**PPV**	**NPV**	**MCC**	**AUC**
RF-Heter	1.000	≤0.001	≤0.001	≤0.001	≤0.001	≤0.001
RF-Hybrid	1.000	0.337	0.337	0.337	0.337	0.134
ABCpred	0.029	0.190	0.043	0.029	0.036	0.085
Bepipred2	1.000	≤0.001	≤0.001	≤0.001	≤0.001	≤0.001
iBCE-EL	≤0.001	0.015	≤0.001	≤0.001	0.008	≤0.001
LBtope	0.127	0.874	0.435	0.099	0.197	0.330
SVMtrip	0.092	0.720	0.232	0.043	0.106	0.029

(recall that the *Hybrid* dataset consists of the joined *OrgSpec* and *Heter* ones), which may contain examples from other related bacteria; from the smaller size of the *OrgSpec* training set in comparison to the other organisms used, which in itself may be sub-optimal for developing a good, generalisable model; from idiosyncrasies of this particular pathogen; or from any of a number of potential causes. Further *in silico* experiments to investigate these potential effects remain to be explored in future works.

Supplementary References 2

O'Loughlin, R. E., Roberson, A., Cieslak, P. R., Lynfield, R., Gershman, K., Craig, A., Albanese, B. A., Farley, M. M., Barrett, N. L., Spina, N. L., Beall, B., Harrison, L. H., Reingold, A., and Beneden, C. V. (2007). The epidemiology of invasive group a streptococcal infection and potential vaccine implications: United states, 2000-2004.
