# Supplementary material for: Organism-specific training improves performance of linear B-cell epitope prediction
Source: Bioinformatics. 2021 Jul 21;37(24):4826–34. doi: 10.1093/bioinformatics/btab536 (PMC8665745; doi:10.1093/bioinformatics/btab536)
Supplement: btab536_Supplementary_Data [file btab536_supplementary_data.zip › 00_2_Supplementary_File_3.pdf]

Supplementary File 3
Supplementary Figures and Captions

July 1, 2021

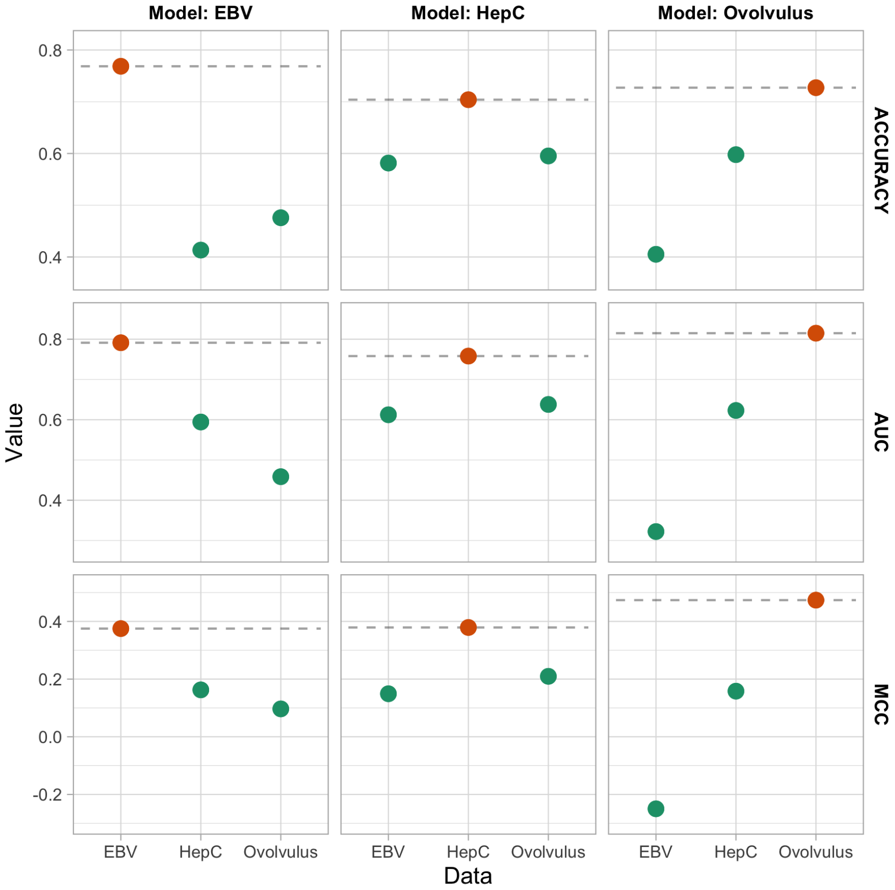

Figure S3 - 1: Aggregated performance indices of each organism-specific model on each organism-specific hold-out set. This shows how the organism-specific training results in models that generalise very well to the particular pathogen for which they were developed, at the cost of degraded performance for other pathogens.

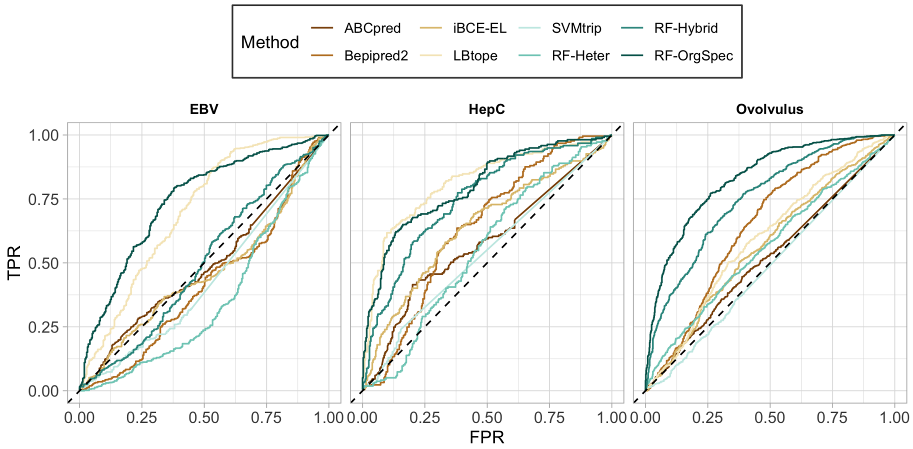

Figure S3 - 2: ROC curves for all predictors tested on the hold out sets for the Epstein-Barr Virus (left), Hepatitis C virus (centre) and *O. volvulus* (right).

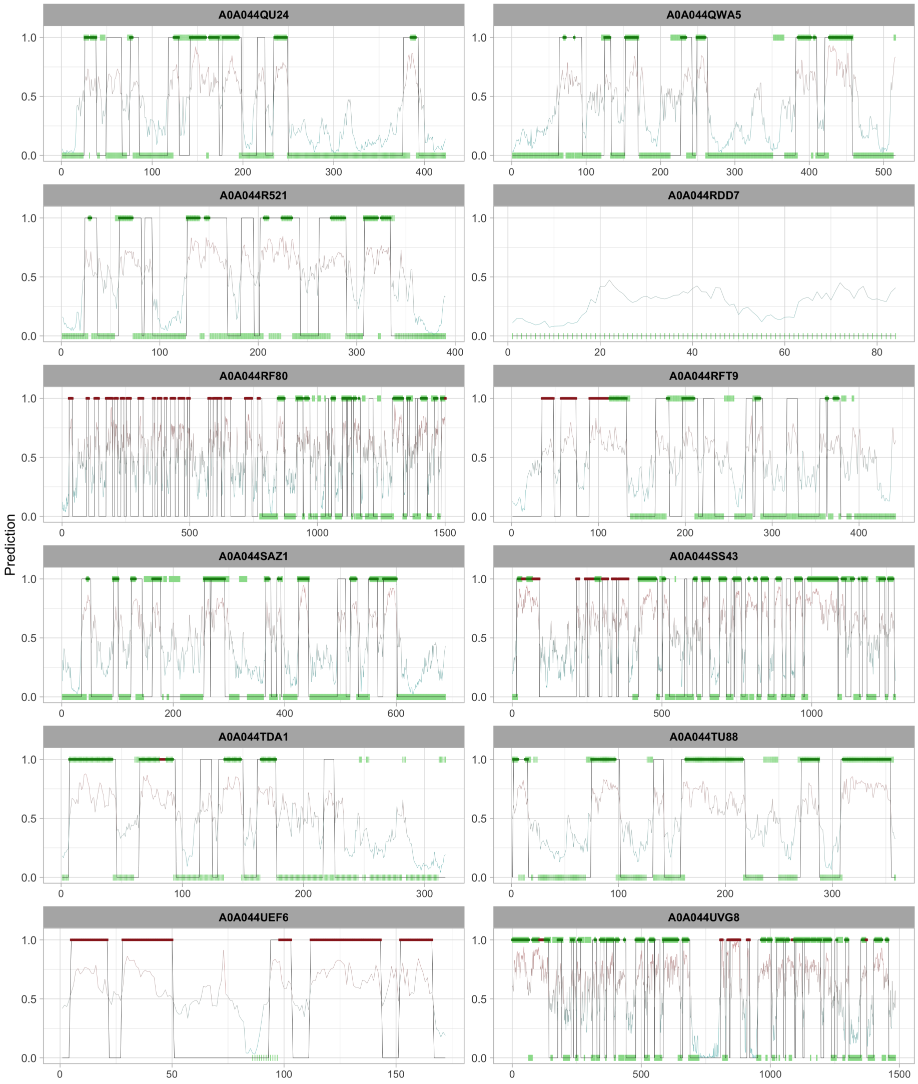

Figure S3 - 3: *RF-OrgSpec* predictions for the proteins on the *O. volvulus* hold-out set, part 1. The narrow line shows the predicted probability returned by *RF-OrgSpec*, which was thresholded to yield positive/negative predictions. Light-green areas indicate regions that were labelled as positive/negative in the IEDB data. Narrow dark green indicates **true positive** predictions, and red indicates new candidate targets, that is, regions without known labels that were predicted as positive by the *RF-OrgSpec* model.

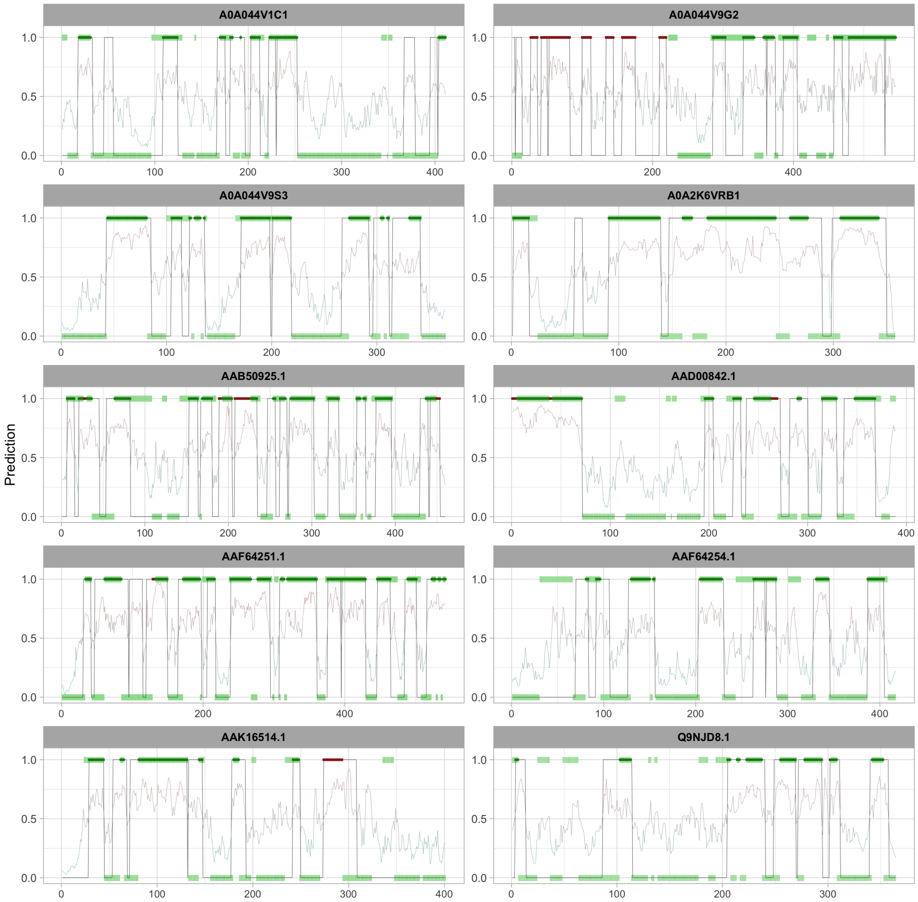

Figure S3 - 4: *RF-OrgSpec* predictions for the proteins on the *O. volvulus* hold-out set, part 2.

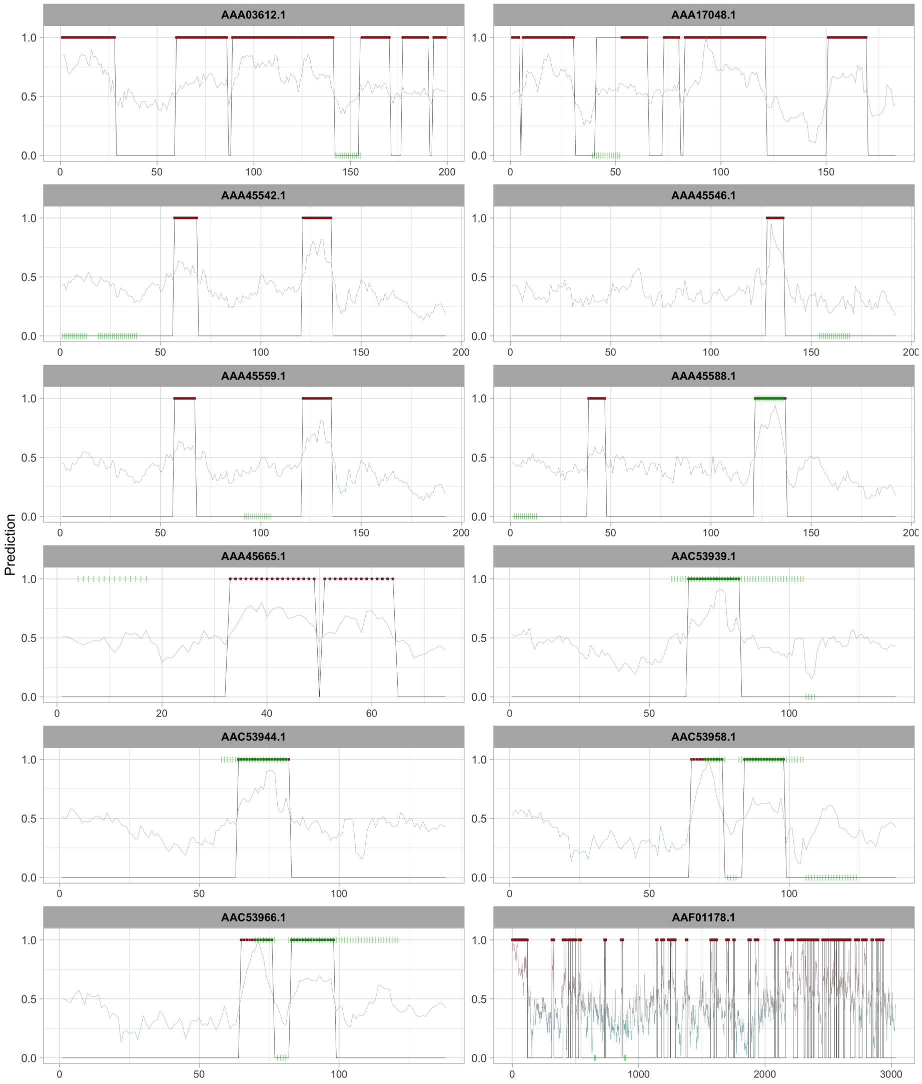

Figure S3 - 5: *OrgSpec* predictions for the proteins on the Hepatitis C hold-out set, part 1. The narrow line shows the predicted probability returned by *OrgSpec*, which was thresholded to yield positive/negative predictions. Light-green areas indicate regions that were labelled as positive/negative in the IEDB data. Narrow dark green indicates **true positive** predictions, and red indicates new candidate targets, that is, regions without known labels that were predicted as positive by the *OrgSpec* model.

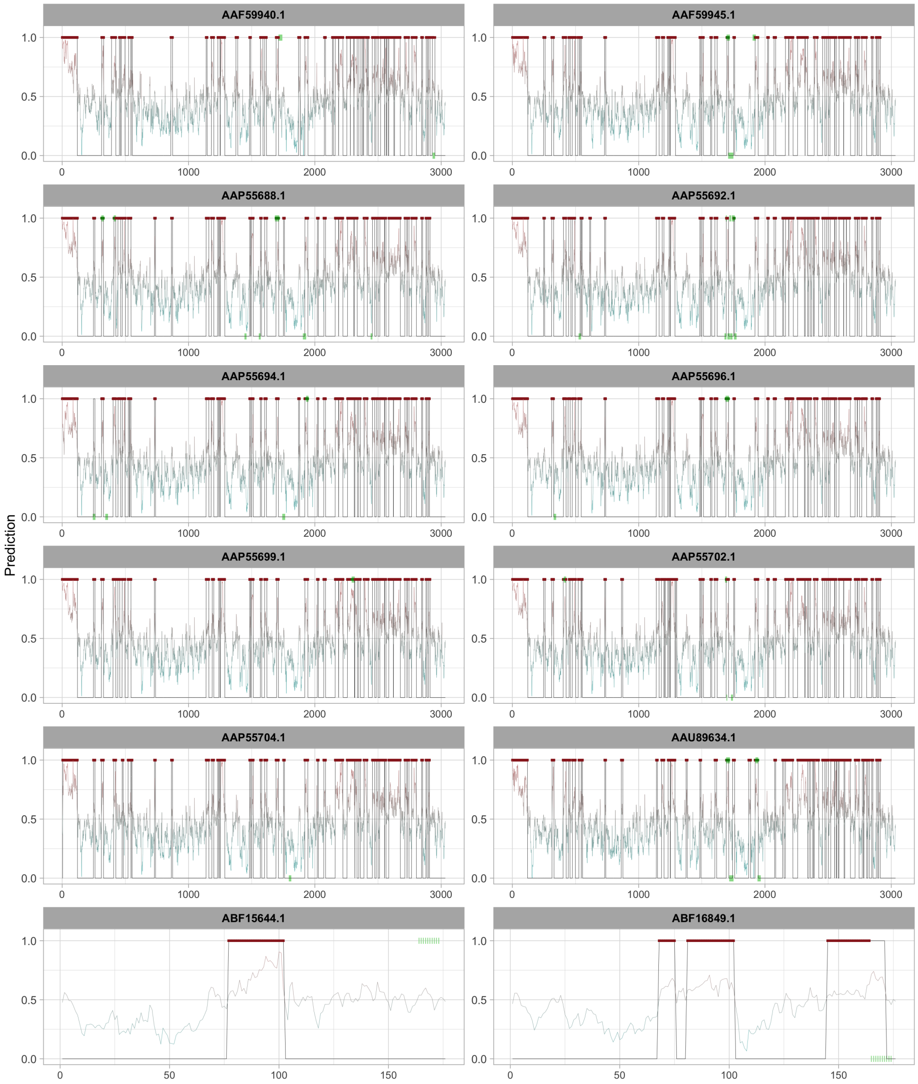

Figure S3 - 6: *OrgSpec* predictions for the proteins on the Hepatitis C hold-out set, part 2.

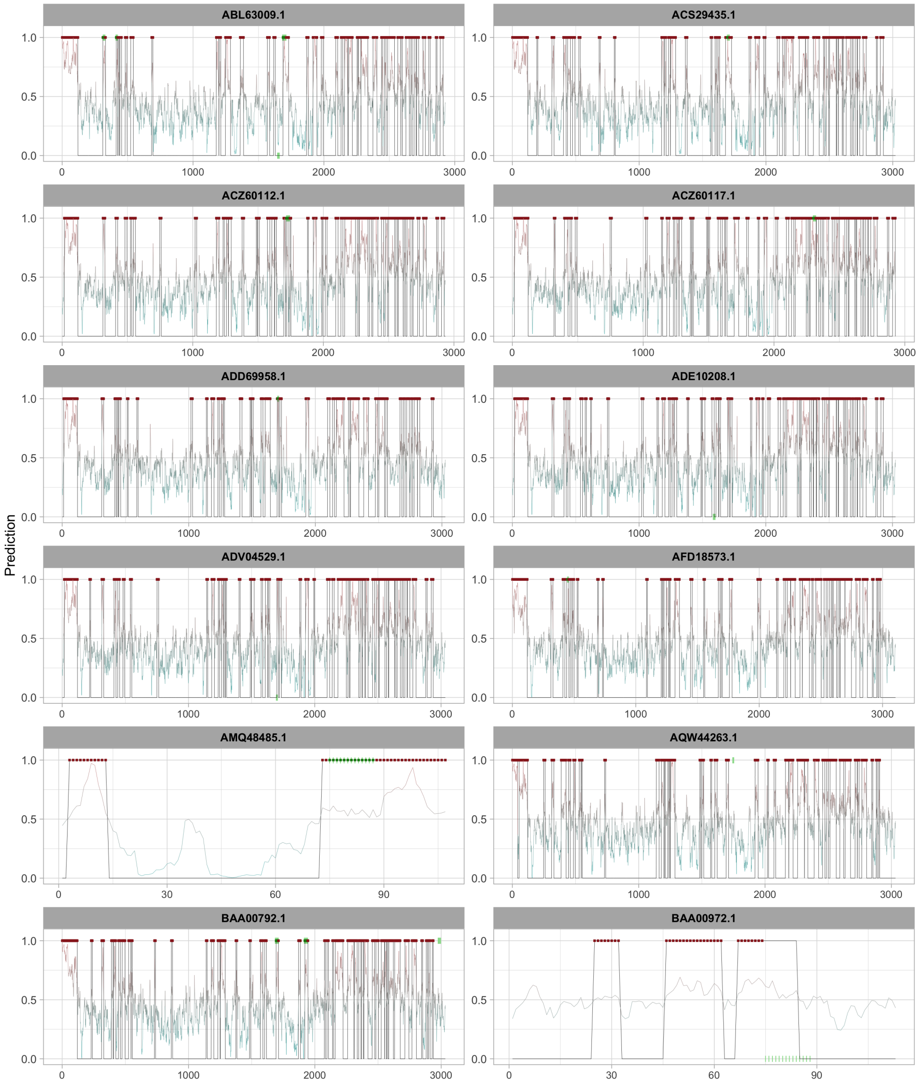

Figure S3 - 7: *OrgSpec* predictions for the proteins on the Hepatitis C hold-out set, part 3.

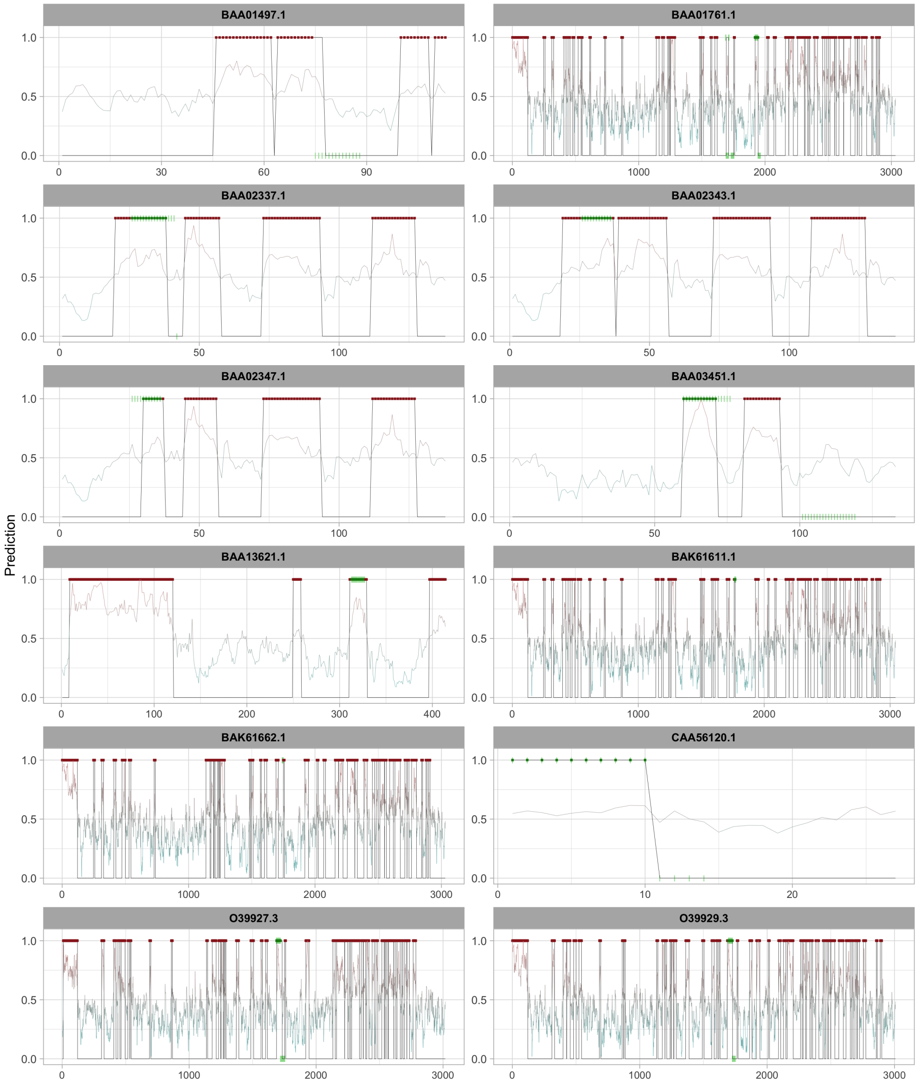

Figure S3 - 8: *OrgSpec* predictions for the proteins on the Hepatitis C hold-out set, part 4.

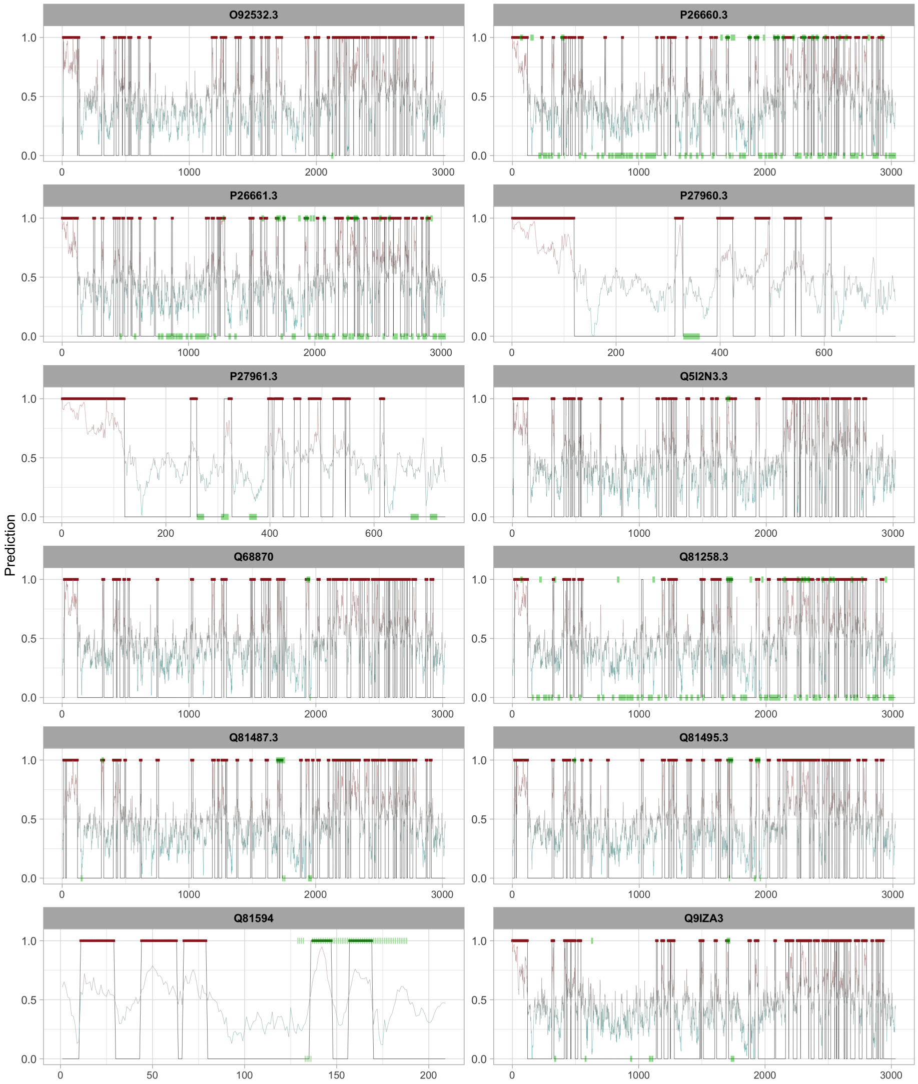

Figure S3 - 9: *OrgSpec* predictions for the proteins on the Hepatitis C hold-out set, part 5.

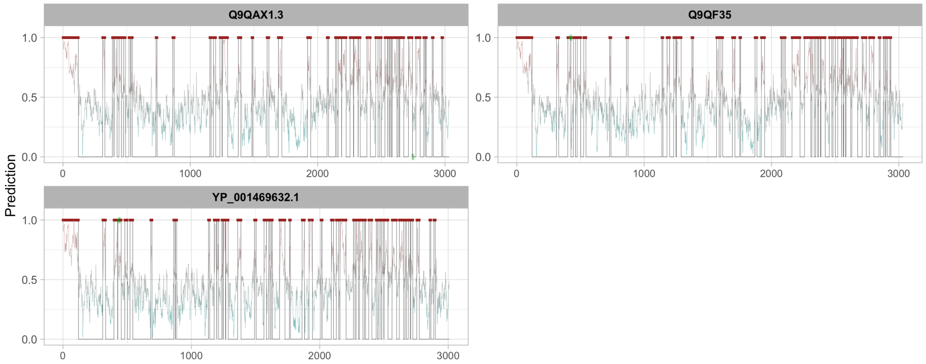

Figure S3 - 10: *OrgSpec* predictions for the proteins on the Hepatitis C hold-out set, part 6.

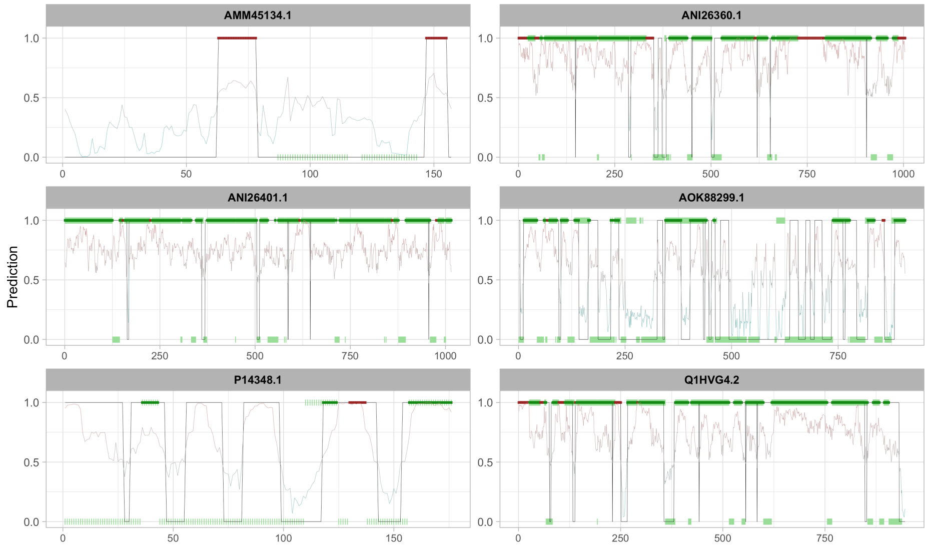

Figure S3 - 11: *OrgSpec* predictions for the proteins on the Epstein-Barr virus hold-out set. The narrow line shows the predicted probability returned by *OrgSpec*, which was thresholded to yield positive/negative predictions. Light-green areas indicate regions that were labelled as positive/negative in the IEDB data. Narrow dark green indicates **true positive** predictions, and red indicates new candidate targets, that is, regions without known labels that were predicted as positive by the *OrgSpec* model.
