# Supplementary material for: Organism-specific training improves performance of linear B-cell epitope prediction
Source: Bioinformatics. 2021 Jul 21;37(24):4826–34. doi: 10.1093/bioinformatics/btab536 (PMC8665745; doi:10.1093/bioinformatics/btab536)
Supplement: btab536_Supplementary_Data [file btab536_supplementary_data.zip › 00_2_Supplementary_File_4.pdf]

Supplementary File 4

July 1, 2021

Model Selection

A variety of classification models were tested, using the *O. volvulus* data set (split into train and test based on protein ID and sequence similarity, and excluding the holdout set used in the main experiments) to investigate model performance for classification problems set in the space of features defined in this work. The following models were tested: Random Forest (RF) (Breiman, 2001) , XGBoost (Chen and Guestrin, 2016), Support Vector Machine (SVM) (Vapnik, 2013) and Multilayer Perceptron Neural Network (MLP) (Haykin, 2004) across multiple performance measures, including Accuracy, Matthews Correlation Coefficient, Area Under the ROC curve and Positive Predictive Value. The model selection and hyper-parameter tuning experiments were run using model implementations available from package Scikit-learn version 0.24.1 (Pedregosa *et al.*, 2011) for all classifiers except XGBoost, for which the implementation available on package (Chen *et al.*, 2019) was employed. All methods were initially run using their default hyper-parameter values, shown in Table 1. The Random Forest model performed the best across all performance measures, as shown in the table 2, and was therefore selected as the modelling approach for all experiments reported in the main paper.

Table S4 - 1: Model hyper-parameters used in the model selection experiments. Default values from each implementation were used.

**Model**	**Parameter: value used**
Random Forest	*n_estimators*: 100
	split criterion: Gini
	max_depth: None
	min_samples: 2
	min_samples_leaf: 1
	min_weight_fraction_leaf: 0
	max_features: auto
	max_leaf_nodes: None
	min_impurity_decrease: 0
	min_impurity_split: None
	bootstrap: True
	warm_start: False
	class_weight: None
	ccp_alpha: 0
	max_samples: None
XGBoost	*eta*: 0.3
	gamma: 0
	max_depth: 6
	min_child_weight: 1
	max_delta_step: 0
	subsample: 1
	sampling_method: auto
	colsample_bytree: 1
	lambda: 1
	alpha: 0
	tree_method: auto
	sketch_eps: 0.03
	scale_pos_weight: 1
	updater: grow_colmaker
	refresh_leaf: 1
	grow_policy: depthwise
	max_leaves: 0
	max_bin: 256
	predictor: auto
Support Vector Machine	*C*: 1
	kernel: rbf
	degree: 3
	gamma: scale
	coef0: 0
	shrinking: True
	probability: True
	tol: 1e-3

Table S4 - 1 – continued from previous page

**Model**	**Parameters: values used (package defaults)**
	cache_size: 200<br>*class_weight*: None<br>*decision_function_shape*: ovr<br>*break_ties*: False
Multilayer Perceptron	*hidden_layers_sizes*: (100,)<br>*activation*: relu<br>*solver*: adam<br>*alpha*: 0.0001<br>*batch_size*: auto<br>*learning_rate*: constant<br>*learning_rate_init*: 0.001<br>*power_t*: 0.5<br>*max_iter*: 200<br>*shuffle*: True<br>*tol*: 1e-4<br>*warm_start*: False<br>*momentum*: 0.9<br>*nesterovs_momentum*: True<br>*early_stopping*: False<br>*validation_fraction*: 0.1<br>*beta_1*: 0.9<br>*beta_2*: 0.999<br>*epsilon*: 1e-8,<br>*n_iter_no_change*: 10<br>*max_fun*: 15000

Table S4 - 2: Point estimates of performance obtained for initial model exploration. ACC = Accuracy, MCC = Matthews Correlation Coefficient, PPV = Positive Predictive Value, AUC = Area Under the ROC Curve.

**Model**	**ACC**	**MCC**	**PPV**	**AUC**
Random Forest	0.695	0.377	0.739	0.753
XGBoost	0.690	0.364	0.727	0.741
Support Vector Machine	0.674	0.334	0.719	0.722
Multilayer Perceptron	0.661	0.300	0.694	0.705

These results motivated the use of Random Forest for the development of all organism-specific models in this paper. Although further, more comprehensive model investigation could be performed, the results obtained exhibited a sufficiently high performance to justify the adoption of RF as our model of choice.

Investigation of Hyper-parameter Tuning

Hyper-parameter tuning was investigated as a possible way to improve the performance of the Random Forest classification model. As in the model selection experiments, we employed the *O. volvulus* data for the tuning experiments. These tests were performed using Scikit-learn's randomised search function, with the following parameters:

- *n_iter*: 200
- *scoring*: mcc_scorer
- *refit*: True
- *error_score*: np.nan
- *pre_dispatch*: None

The hyper-parameters that were tuned for the model were:

- *bootstrap* $\in \{False, True\}$. **Selected value:** True
- *max_features* $\in \{auto, sqrt\}$. **Selected value:** auto
- *min_samples_split* $\in \{10, 15, 20, 25, 30\}$. **Selected value:** 20
- *n_estimators* $\in \{500, 550, 600, \ldots, 1000\}$. **Selected value:** 650
- *min_samples_leaf* $\in \{2, 3, 4, 5, 6\}$. **Selected value:** 4
- *max_depth* $\in \{20, 40, \ldots, 120\}$. **Selected value:** 80

The final performance values obtained after tuning, are shown in Table 3. The baseline results obtained by the standard configuration are also repeated in this table for convenience.

Table S4 - 3: Random Forest results after parameter tuning

**Method**	**ACC**	**MCC**	**PPV**	**AUC**
Benchmark	0.695	0.377	0.739	0.753
After Tuning	0.703	0.394	0.744	0.760

Since hyper-parameter tuning did not seem to substantially affect the end performance of the Random Forest models, the end solution implemented for the experiments in this paper was to not employ tuning for the model hyper-parameters in our final implementation, based on a lowest-complexity approach to pipeline design and in the rationale that the main objective of the paper was to demonstrate the organism-specific training principle, rather than explore model optimisation.

Investigation of Dimensionality Reduction

A selection of dimensionality reduction techniques were investigated for the epitope prediction problem, including: (i) filter methods based on extracting the top K features, using both Mutual Information (Vergara and Estévez, 2014) and the Anova F-value (Montgomery and Runger, 2003) as ordering scores; (ii) Principal Component Analysis (PCA) (Tan *et al.*, 2005); and (iii) a wrapper method, Maximum Relevance-Minimum Redundancy (MRMR) (Peng *et al.*, 2005). Those methods were tested using the the *O. volvulus* data set (split into train and test based on protein ID and sequence similarity, and excluding the holdout set used in the main experiments). Scikit-learn implementations were used for all techniques, and all methods were tested using their default parameters,shown in Table 4.

Table S4 - 4: Default dimensionality reduction technique parameters

**Method**	**Parameters**
Principal Component Analysis	*n_components*$\in \{0.95, 0.5, 0.15\}$<br>*copy*: True<br>*whiten*: False<br>*sdv_solver*: auto<br>*tol*: 0<br>*iterated_power*: auto
Mutual Information	*discrete_features*: auto<br>*n_neighbors*: 3<br>*copy*: True
Select K Best	*score_func*: f_classif<br>*k*: 15
MRMR	*K*: 15<br>*relevance*: f<br>*redundancy*: c<br>*denominator*: mean<br>*only_same_domain*: False

None of the reduced feature sets resulted in significant performance gains for any of the classifiers tested (see Table 1). As an illustrative example, Table 5 shows the results for the default Random Forest classifier.

Since the main focus of this work was in demonstrating the effects of organism-specific training rather than developing a fully-deployed pipeline, we opted (based on the observed lack of improvement) to retain the full feature sets in the solutions developed in the main manuscript, since feature reduction did not yield any observable generalisation performance gains and the fact that Random Forest already performs its own embedded feature prioritisation process. Reducing the feature space will, however, be useful when implementing a user-facing interface for the organism-specific pipeline, as it can reduce the computational costs associated with feature calculation and model fitting.

Table S4 - 5: Results after dimensionality reduction on the performance of the default RF classifier. The values next to PCA refer to the proportion of variance retained. The baseline values obtained by the Random Forest on the full feature set are repeated here for convenience (referenced as *benchmark*).

**Method**	**Features**	**ACC**	**MCC**	**PPV**	**AUC**
Benchmark	845	0.695	0.377	0.739	0.753
Mutual Information	15	0.688	0.365	0.710	0.746
PCA (0.95)	521	0.655	0.284	0.683	0.699
PCA (0.50)	121	0.674	0.334	0.718	0.726
PCA (0.15)	11	0.678	0.353	0.740	0.734
Select K Best	70	0.691	0.370	0.736	0.745
MRMR	15	0.700	0.385	0.738	0.747

Supplementary References 4

Breiman, L. (2001). Random forests. *Machine learning*, **45**(1), 5–32.

Chen, T. and Guestrin, C. (2016). XGBoost: A scalable tree boosting system. In *Proceedings of the 22nd ACM SIGKDD International Conference on Knowledge Discovery and Data Mining*, KDD '16, pages 785–794, New York, NY, USA. ACM.

Chen, T., Okhlopkov, D., Cho, P., and Turkov, M. (2019). Xgboost extreme gradient boosting.

Haykin, S. (2004). *Neural Networks: A Comprehensive Foundation*. Prentice Hall, 2nd edition.

Montgomery, D. C. and Runger, G. C. (2003). *Applied Statistics and Probability for Engineers*. John Wiley and Sons.

Pedregosa, F., Varoquaux, G., Gramfort, A., Michel, V., Thirion, B., Grisel, O., Blondel, M., Prettenhofer, P., Weiss, R., Dubourg, V., Vanderplas, J., Passos, A., Cournapeau, D., Brucher, M., Perrot, M., and Duchesnay, E. (2011). Scikit-learn: Machine learning in Python. *Journal of Machine Learning Research*, **12**, 2825–2830.

Peng, H., Long, F., and Ding, C. (2005). Feature selection based on mutual information criteria of max-dependency, max-relevance, and min-redundancy. *IEEE Transactions on pattern analysis and machine intelligence*, **27**(8), 1226–1238.

Tan, P.-N., Steinbach, M., and Kumar, V. (2005). *Introduction to Data Mining*. Addison Wesley.

Vapnik, V. (2013). *The nature of statistical learning theory*. Springer science & business media.

Vergara, J. R. and Estévez, P. A. (2014). A review of feature selection methods based on mutual information. *Neural computing and applications*, **24**(1), 175–186.
