# Supplementary material for: Organism-specific training improves performance of linear B-cell epitope prediction
Source: Bioinformatics. 2021 Jul 21;37(24):4826–34. doi: 10.1093/bioinformatics/btab536 (PMC8665745; doi:10.1093/bioinformatics/btab536)
Supplement: btab536_Supplementary_Data [file btab536_supplementary_data.zip › 00_2_Supplementary_File_5.pdf]

Supplementary File 5

July 1, 2021

Performance indicators

Several performance indicators were calculated to provide comparability with different references in the literature, and to explore distinct aspects of the predictive behaviour of the models. In the definitions below, TP, TN, FP and FN denote the counts of True Positives, True Negatives, False Positives and False Negatives, respectively.

- **Positive Predictive Value**,

  $$PPV = \frac{TP}{TP + FP}.$$

  Quantifies the probability of a peptide actually representing an epitope, given that it was classified as *positive*. This measure provides an indication of how trustworthy the positive predictions of each model are, which is relevant for justifying the application of resources to the experimental validation of the predicted targets. PPV is also referred to as *Precision*.

- **Negative Predictive Value**,

  $$NPV = \frac{TN}{TN + FN}.$$

  Quantifies the probability of a peptide not representing an epitope, given that it was classified as *negative*. This measure provides an indication of how assertive the model is when not recommending a peptide, i.e., how likely it is not to miss potentially valuable targets.

- **Sensitivity**,

  $$SENS = \frac{TP}{TP + FN}.$$

  Measures the probability of correctly identifying an epitope (positive class) from the data. Sensitivity is also known as the **True Positive Rate** (TPR).

- **Accuracy**,

$$ACC = \frac{TP + TN}{TP + FP + TN + FN}.$$

Quantifies the overall rate of correct classifications. Although it represents a global performance index, accuracy is sensitive to class imbalance and implicitly assumes equal misclassification costs for false positives and false negatives, which is not necessarily the case for epitope prediction.

- **Matthews Correlation Coefficient**,

$$MCC = \frac{TP \times TN - FP \times FN}{\sqrt{(TP + FP)(TP + FN)(TN + FP)(TN + FN)}}.$$

A combined performance index that "generates a high score only if the binary predictor was able to correctly predict the majority of positive data instances and the majority of negative data instances" (Chicco and Jurman, 2020). The MCC provides a unified metric for the comparison of the overall performance of the predictors.

- **Area under the ROC curve** (AUC), which quantifies the robustness of the trade-off between the Sensitivity and the False Positive Rate (FPR),

$$FPR = \frac{FP}{FP + TN}$$

of a classification model, under different classification thresholds.

Supplementary References 5

Chicco, D. and Jurman, G. (2020). The advantages of the matthews correlation coefficient (MCC) over f1 score and accuracy in binary classification evaluation. *BMC Genomics*, **21**(1).
