# Supplementary material for: Organism-specific training improves performance of linear B-cell epitope prediction
Source: Bioinformatics. 2021 Jul 21;37(24):4826–34. doi: 10.1093/bioinformatics/btab536 (PMC8665745; doi:10.1093/bioinformatics/btab536)
Supplement: btab536_Supplementary_Data [file btab536_supplementary_data.zip › 00_2_Supplementary_File_6.pdf]

Supplementary File 6

July 1, 2021

Feature Relevance

In an attempt to understand the possible reasons behind the improved performance of models trained on organism-specific data, we have conducted a graphical investigation of feature relevance, as returned by the Random Forest models fit on both organism-specific and heterogeneous sets. This section documents this exploration, with the figure captions indicating points of particular interest. In all figures, the feature families are referred as follows (notice that all features are calculated based on the local 15 aminoacid-wide neighbourhood of each position of a protein sequence. Please refer to Section 2.1.1 of the manuscript for details):

- **AAdescr.**: 66 physiochemical descriptors of amino acid residues .
- **Atoms**: Count of Carbon, Hydrogen, Nitrogen, Oxygen and Sulphur atoms.
- **CT**: 343 conjoint triad frequencies.
- **Entropy**: Sequence entropy.
- **Freq-1AA**: Frequencies of occurrence of each amino-acid.
- **Freq-2AA**: Frequencies of occurrence of each dipeptide.
- **Freq-Types**: Frequencies of occurrence of each of the 9 amino-acid types (aliphatic, hydrophobic, etc.)
- **Mol. weight**: Molecular weight.

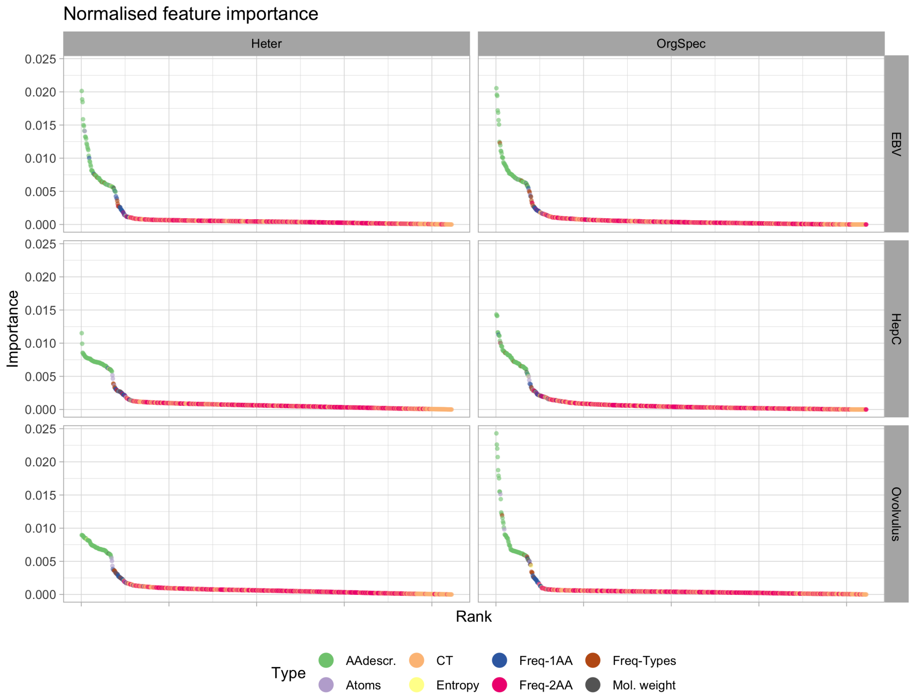

Figure S6 - 1: Feature importance (as calculated by the RF models), coloured by feature family. Notice that *Freq-2AA* and *CT* features contribute almost nothing to the predictive ability of RF models in all cases, and that the *AAdescr*-type features are consistently selected as the most relevant by Random Forest.

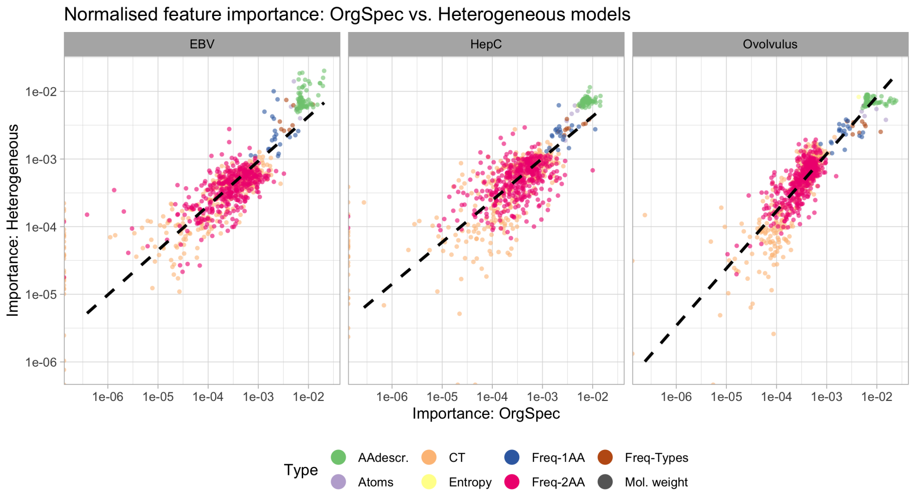

Figure S6 - 2: Feature importance for organism-specific vs. heterogeneous data-trained models, stratified by pathogen. The dashed regression line provides a reference for qualitatively assessing the high correlation (notice that both axes are log-scaled). This again highlights the high relevance of the physiochemical descriptors ($AAdescr$) , followed by the atom counts and single-aminoacid frequencies. Dipeptide and CT frequencies do not contribute significantly to any of the models.

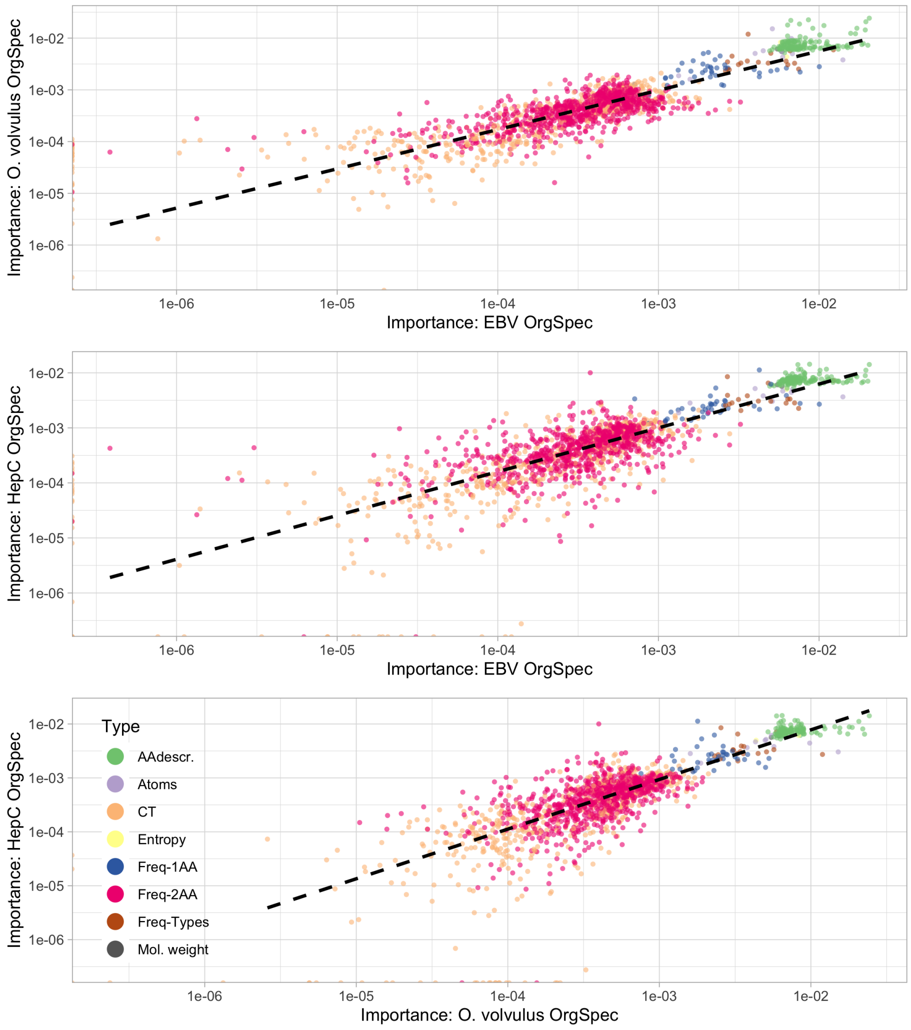

Figure S6 - 3: Feature importance for different organism-specific models. The dashed regression line provides a reference for qualitatively assessing the high correlation (notice that both axes are log-scaled). The same patterns of relative importance of feature families observed in figure 3 are also observed here.

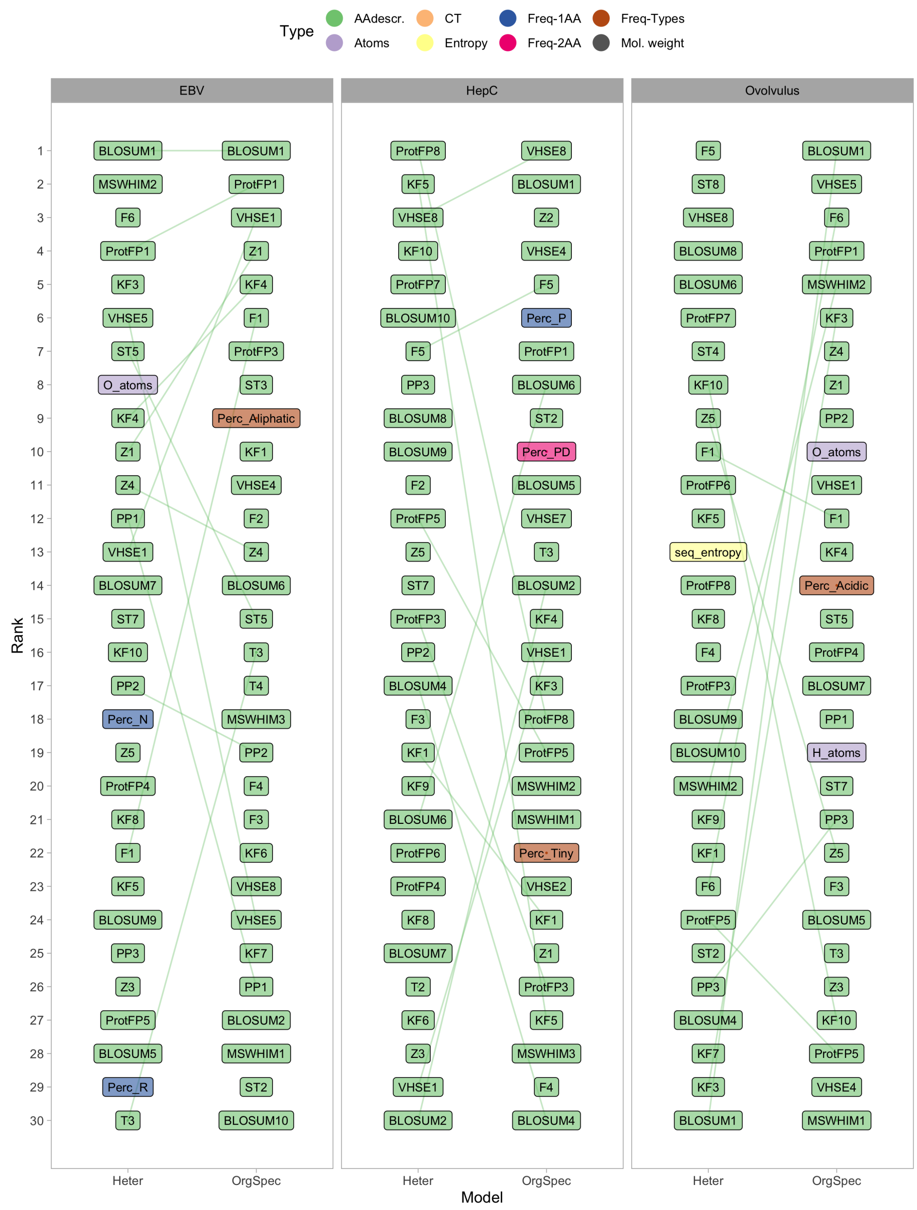

Figure S6 - 4: Thirty most highly relevant features according to each Random Forest model trained for each pathogen (organis-specific and heterogeneous). The high prevalence of *AAdescr* features is clear, although they make up only a fraction of the total feature space (66/845 features ≊ 7.8%).

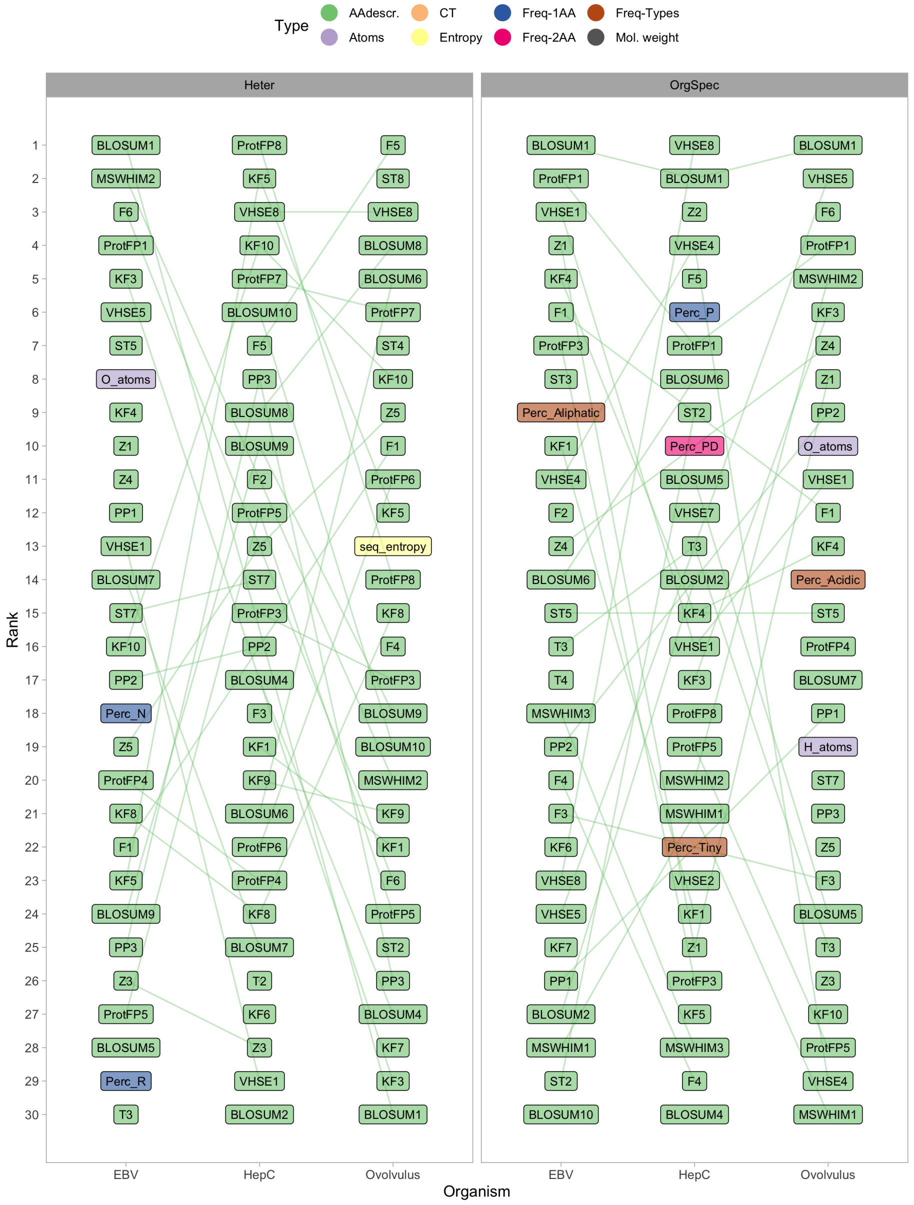

Figure S6 - 5: Another visualisation of the thirty most relevant features used by the Random Forest models. Notice how feature BLOSUM1 (Georgiev, 2009) appears as particularly relevant for all organism-specific models. Although interesting, the detailed investigation of underlying mechanisms potentially represented by this particular feature is beyond the scope of this work.

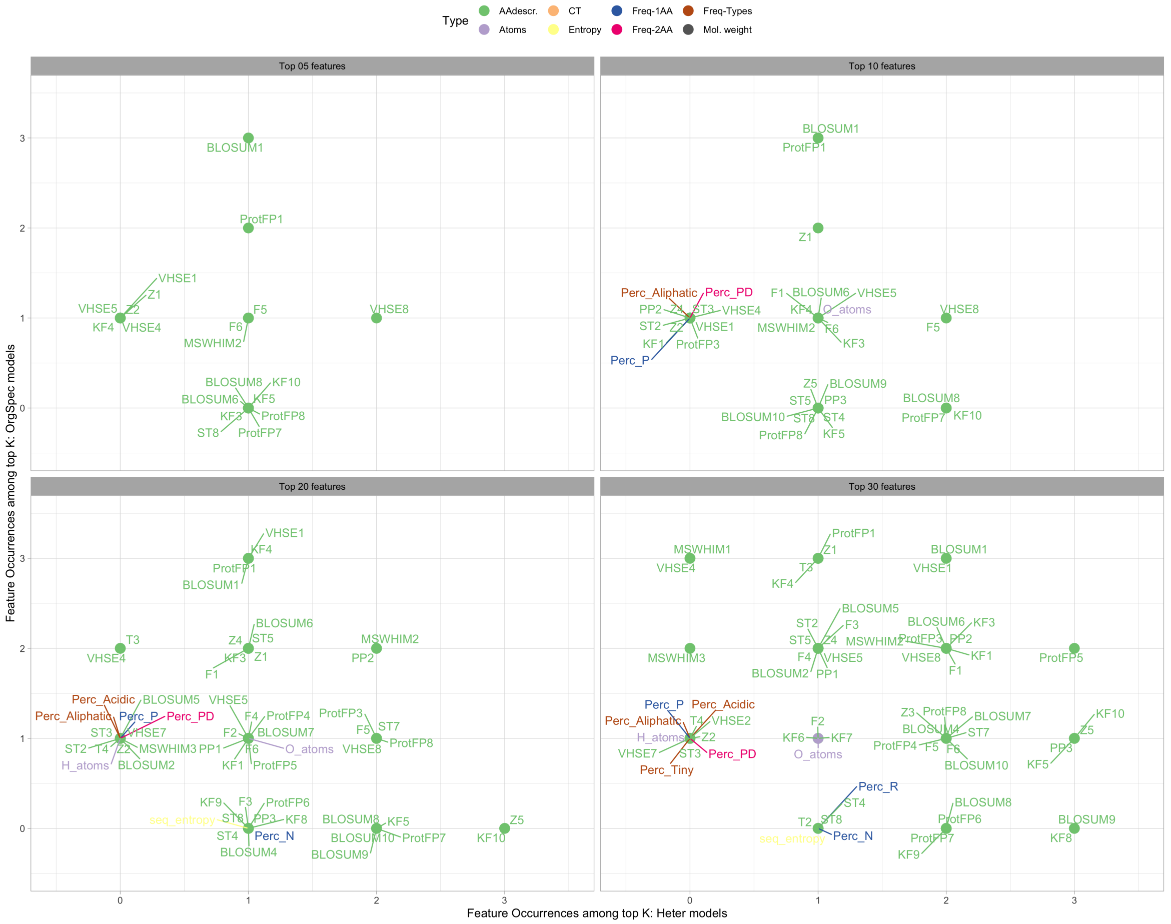

Figure S6 - 6: Number of times features appear among the top 5, 10, 20 and 30 features, for the Heterogeneous and Organism-specific models (max = 3). Notice how feature *BLOSUM1* shows a high relevance for the Organism-specific models (see also Figure 5). This feature is strongly associated with hydrophobicity, with an $r^2 = 0.94$ according to (Georgiev, 2009). In the case of the data representation used in this work, it measures the average hydrophobicity of the 15-AA neighbourhood of a given position on the protein.

Spatial distribution of observations

To explore the neighbourhood structure of the datasets, we have employed t-SNE projections (Van der Maaten and Hinton, 2008). The purpose of this exploration was to (i) investigate whether data coming from distinct pathogens present different clustering structures in terms of positive/negative observations; or (ii) whether data coming from different pathogens is located in distinct regions of the space of features. Either case would help explain the improved performance of the organism-specific models when compared to generalist approaches, from a modelling perspective (albeit not necessarily in terms of underlying biological mechanisms). These explorations are illustrated in Figure 7 below, as well as in Figure 3 of the main text.

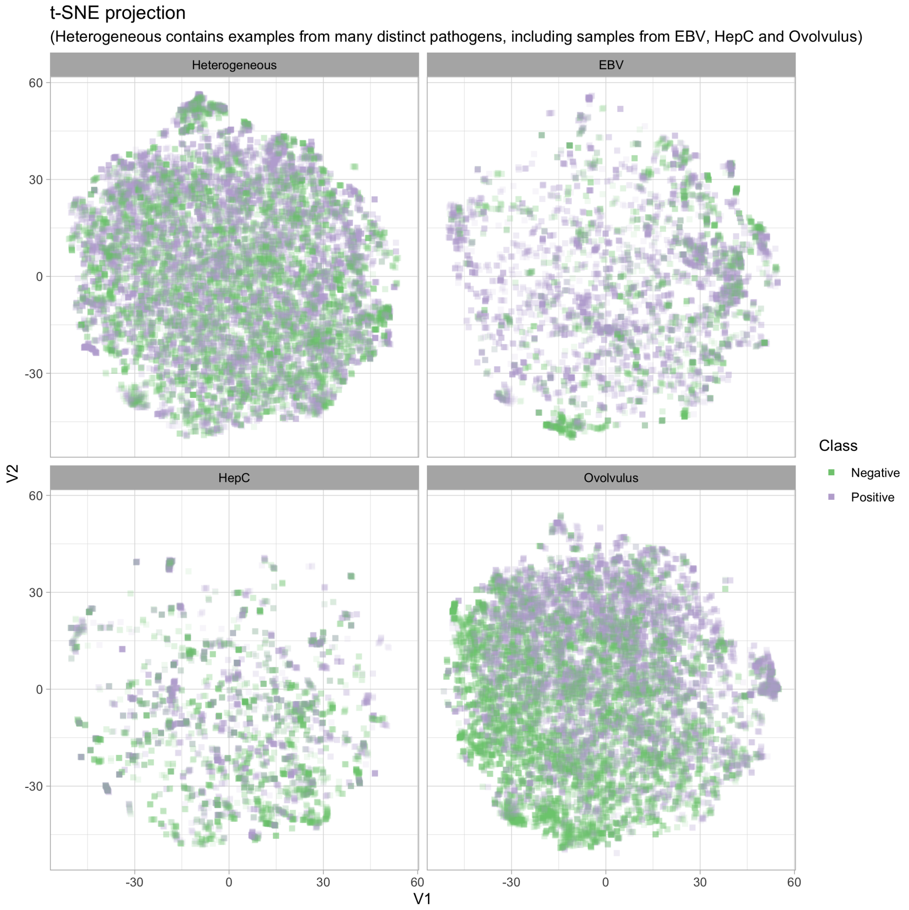

Figure S6 - 7: t-SNE projection of the data. The projection was calculated on the entirety of the data, and later split by pathogen. Notice how heterogeneous data (which is composed of observations from many different organisms) has positive and negative points distributed almost uniformly over the projected space. Contrast that with the organism-specific data, for which different regions of the space exhibit distinct balances of positive/negative observations, which potentially makes the fitting of classification models more capable of partitioning the feature space into more homogeneous sub-regions. See also Figure 3 in the main text. It is also apparent that distinct regions of the projected space are more or less occupied by data coming from different pathogens (contrast, e.g., the density of points above $V2 = 30$ between *O. volvulus* and $HepC$).

Supplementary References 6

Georgiev, A. G. (2009). Interpretable numerical descriptors of amino acid space. *Journal of Computational Biology*, **16**(5), 703–723.

Van der Maaten, L. and Hinton, G. (2008). Visualizing data using t-sne. *Journal of machine learning research*, **9**(11).
